# Computational Design of Cyclic Peptide Inhibitors of a Bacterial Membrane Lipoprotein Peptidase

**DOI:** 10.1021/acschembio.4c00076

**Published:** 2024-05-07

**Authors:** Timothy W. Craven, Mark D. Nolan, Jonathan Bailey, Samir Olatunji, Samantha J. Bann, Katherine Bowen, Nikita Ostrovitsa, Thaina M. Da Costa, Ross D. Ballantine, Dietmar Weichert, Paul M. Levine, Lance J. Stewart, Gaurav Bhardwaj, Joan A. Geoghegan, Stephen A. Cochrane, Eoin M. Scanlan, Martin Caffrey, David Baker

**Affiliations:** † Department of Biochemistry, University of Washington, Seattle, Washington 98195, United States; ‡ Institute for Protein Design, University of Washington, Seattle, Washington 98195, United States; § School of Chemistry, Trinity College Dublin, Dublin D02 R590, Ireland; ∥ School of Medicine and School of Biochemistry and Immunology, Trinity College Dublin, Dublin D02 R590, Ireland; ⊥ Biological Inorganic Chemistry Laboratory, The Francis Crick Institute, London NW1 1AT, U.K.; # School of Chemistry and Chemical Engineering, Queen’s University Belfast, David Keir Building, Stranmillis Road, Belfast BT9 5AG, U.K.,; ∇ Department of Microbiology, Moyne Institute of Preventive Medicine, School of Genetics and Microbiology, Trinity College Dublin, Dublin D02 VF25, Ireland; ○ Institute of Microbiology and Infection, College of Medical and Dental Sciences, University of Birmingham, Birmingham B15 2TT, U.K.; ◆ Howard Hughes Medical Institute, University of Washington, Seattle, Washington 98195, United States

## Abstract

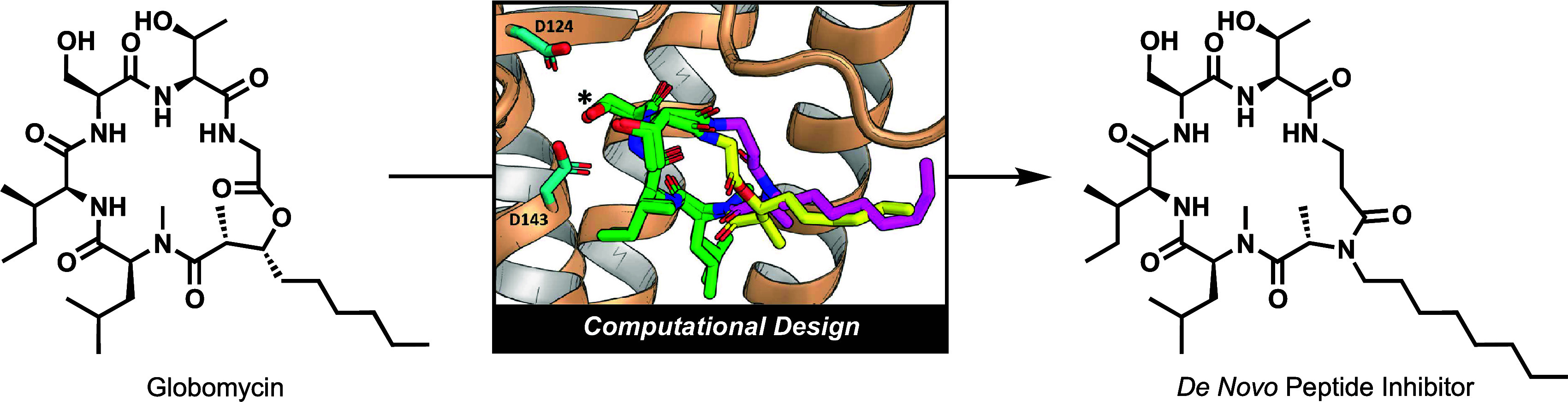

There remains a critical need for new antibiotics against multi-drug-resistant Gram-negative bacteria, a major global threat that continues to impact mortality rates. Lipoprotein signal peptidase II is an essential enzyme in the lipoprotein biosynthetic pathway of Gram-negative bacteria, making it an attractive target for antibacterial drug discovery. Although natural inhibitors of LspA have been identified, such as the cyclic depsipeptide globomycin, poor stability and production difficulties limit their use in a clinical setting. We harness computational design to generate stable *de novo* cyclic peptide analogues of globomycin. Only 12 peptides needed to be synthesized and tested to yield potent inhibitors, avoiding costly preparation of large libraries and screening campaigns. The most potent analogues showed comparable or better antimicrobial activity than globomycin in microdilution assays against ESKAPE-E pathogens. This work highlights computational design as a general strategy to combat antibiotic resistance.

Antibiotic resistance represents a major threat to public health and has spurred significant research into the development of new antimicrobials for the treatment of bacterial infections.^1,2^ Central to this objective is the identification of targets and lead compounds, which often undergo lengthy optimization processes.^3^ Lead compounds can be based on natural products, identified by screening large compound libraries, or generated by machine learning^4^ and genome mining.^5−7^ Despite the need for new antibiotics, there have been few newly approved drugs for clinical use, and attrition rates in antibiotic discovery are high, drastically increasing the associated costs.^8^ New and more efficient tools for developing potent and stable drug candidates are urgently needed.

The bacterial lipoprotein (BLP) biosynthesis pathway represents an attractive antimicrobial target as the enzymes essential for BLP post-translational processing are located in the cytoplasmic membrane with active sites facing the periplasm.^9−12^ In the first step of this pathway, the pre-proBLP (ppBLP) is lipid-modified by the enzyme lipoprotein diacylglyceryl transferase (Lgt), forming the corresponding proBLP (pBLP). Next, lipoprotein signal peptidase II (LspA) cleaves the signal peptide (SP) from the pBLP, producing a diacylated BLP (DA-BLP) that is further lipidated by lipoprotein *N*-acyltransferase (Lnt) for trafficking to the outer membrane in Gram-negative bacteria (Figure 1). The second enzyme in the pathway, LspA, is essential for bacterial viability. Inhibition of this peptidase by the natural products globomycin^13^ and myxovirescin^14^ has been shown to induce bacterial cell death, establishing it as a promising target for combating multidrug resistance.

**Figure 1:**
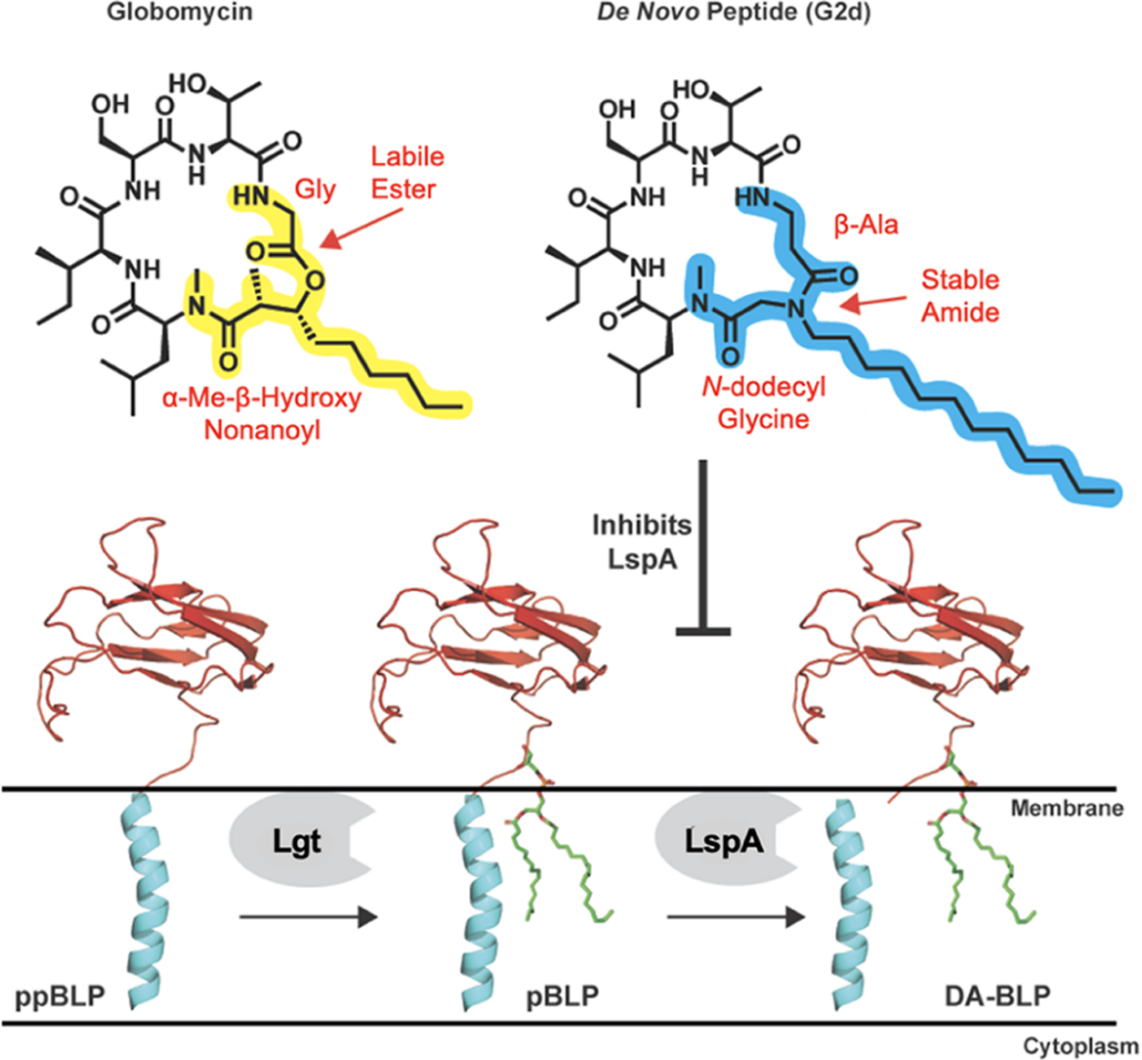
Structure of globomycin, a *de novo* designed peptide, and the first two steps of the BLP biosynthetic pathway. Globomycin inhibits cleavage of the SP (cyan) from a pBLP by LspA.

Globomycin is a 6-residue, cyclic depsipeptide that incorporates a (*2R*,*3R*)-3-hydroxy-2-methylnonanoic moiety into the ring *via* amide and ester linkages. It is produced by strains of the Gram-positive bacteria *Streptomyces*.^15^ Although globomycin shows promising antibacterial activity, poor stability and production difficulties limit its application in the clinic.^16^ Extensive efforts have been devoted to developing a total synthesis of globomycin that involved the use of multiple synthetically accessed building blocks with strict chirality requirements.^17,18^ Further, the poor *in vivo* stability of globomycin limits its application.^19^ SAR studies have produced moderately active analogues in a process requiring the time-consuming synthesis of a large number of compounds. It has been found that variation of the hydrophobic region or replacement of the ester linkage leads to reduced potency,^18,20−22^ limiting the development of more stable analogues.

We have developed general methods for the computational design of peptide macrocycles that can adopt a single stable and biologically active conformation,^23,24^ which have shown promise for the design of peptidic inhibitors of therapeutic targets. Here, we set out to use this computational design method to generate *de novo* cyclic peptide analogues of globomycin, in which the labile depsipeptide ester moiety is replaced by a more stable amide linkage.

Prior to initiating computational design, we first explored three primary variables: cyclic vs linear structures, macrocycle size, and lipid length (Figure S1). Where desired, cyclization was achieved through amide bond formation or ring-closing metathesis (Figures S2–S4). In addition to linear analogues (S1–S4), analogues with lipidic components of varying length (S5–S10), varying macrocycle sizes (S11–S13), and varying stereochemistry (S14–S16) were synthesized. Despite the many analogues generated, this approach furnished compounds showing little or no inhibition of LspA from *Pseudomonas aeruginosa* (*Pa*LspA) (Supplementary discussion).

We next turned to computational peptide design to develop globomycin mimetics featuring the replacement of the ester in globomycin with a more durable amide (Figures 1 and 2), seeking to maintain both the macrocycle structure and the chemical affinity for LspA binding. We explored a range of canonical and non-canonical amino acids to replace the depsipeptide segment, aiming to identify sequences that would closely reproduce the overall shape and functionality of globomycin. We specifically targeted sequences predicted to fold in a manner recapitulating the structure of the *N*-(methyl)-L-leucine, allo-L-isoleucine, L-serine, and allo-L-threonine segments in the LspA-globomycin complex crystal structure (PDB ID 5DIR),^15^ while also enhancing interactions with LspA. Designed sequences that were confidently predicted by Rosetta (Pnear value of 0.6^23^) to generate structures with these properties were selected for synthesis.

**Figure 2:**
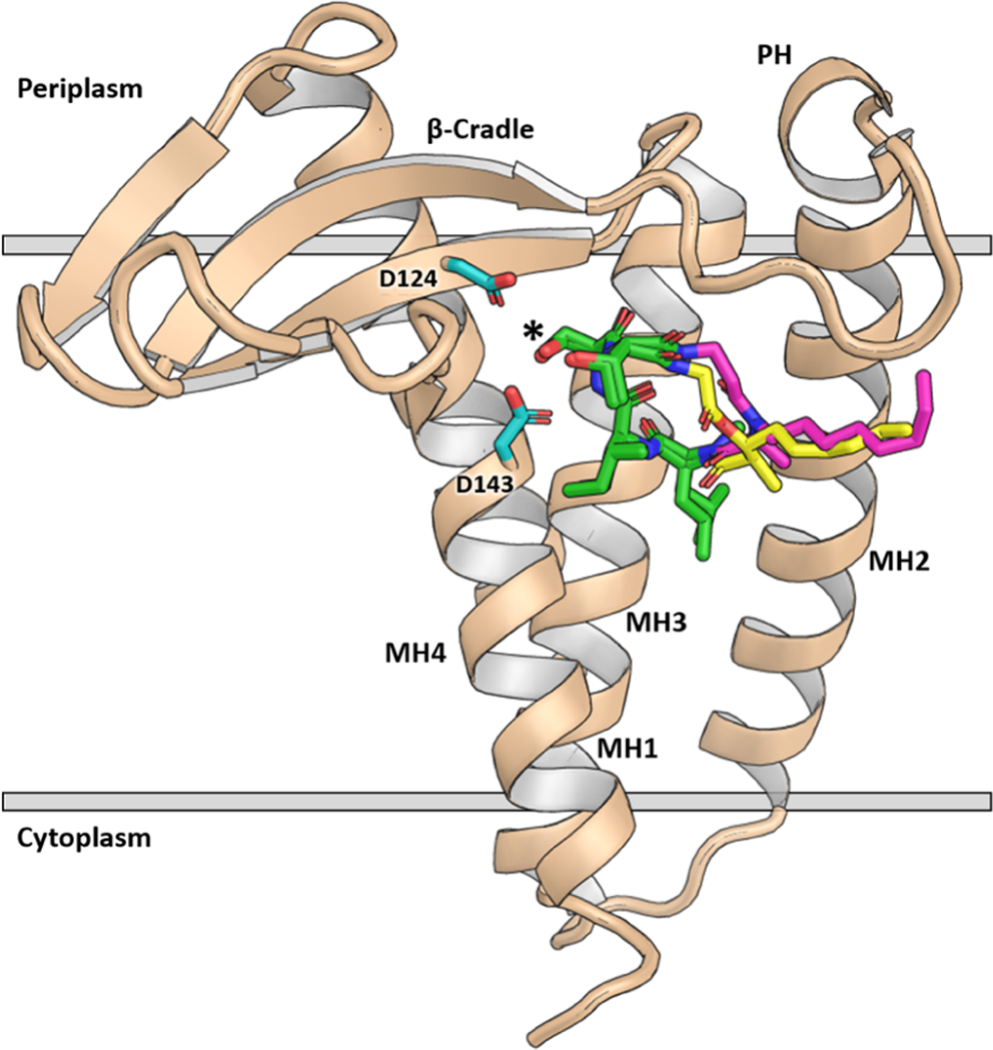
Aligned structure of globomycin bound and **G2a** docked to *Pa*LspA. The labile depsipeptide portion of globomycin is colored yellow, and the β-Ala residue of **G2a** with an amide linkage is colored magenta. Catalytic aspartate dyad residues D124 and D143 are shown in cyan. The blocking hydroxyl of globomycin and **G2a** is labeled with an asterisk. Gray horizontal lines represent approximate membrane boundaries.

In the first generation (Gen1), ten compounds were identified, and six were successfully synthesized (**G1a**-**f**) (Figure S5). The overall structure of these compounds was somewhat conserved, with variations in stereochemistry at the lipid and β-amino acid. While compounds **G1a** and **G1b** contained β-alanine (β-Ala), and were stereochemically different at the α position of the lipid chain, the others (**G1c**, **G1d**, **G1e**, and **G1f**) were further substituted with β-homoalanine (β-hAla). Relative to globomycin, the 19-atom macrocycle was maintained by using the β-amino acid. This facilitated the use of N-alkylated glycine for macrocyclization *via* an amide bond, which replaced the labile ester linkage. Importantly, the aforementioned residues of globomycin were conserved.

These analogues were tested for activity against *Pa*LspA using an *in vitro* fluorescence resonance energy transfer (FRET) activity assay.^21^ LspA inhibition was detected by a change in fluorescence upon cleavage of a FRET peptide substrate containing an N-terminal quencher moiety and a C-terminal fluorophore. All six compounds inhibited *Pa*LspA with IC_50_ values between 2.9 and 9.5 μM (Figures S6 and S7). The two most potent compounds were the stereoisomers **G1a** and **G1b**, with IC_50_ values of 2.94 ± 0.85 and 3.68 ± 0.42 μM, respectively. Globomycin exhibited an IC_50_ value of 40 nM. The change in stereochemistry between analogues **G1e** (IC_50_ 3.56 ± 0.25 μM) and **G1f** (IC_50_ 8.89 ± 0.55 μM) induced a difference in inhibitory activity. Similarly, for **G1c** (IC_50_ = 6.04 ± 0.71 μM) and **G1d** (IC_50_ = 9.48 ± 0.60 μM), the **G1c** analogue with *S* stereochemistry displayed more potent inhibition. Of the three pairs of analogues, the *S* stereocenter at the lipid component produced stronger inhibition.

Based on the structures of the most potent Gen1 analogues **G1a** and **G1b**, a second round of computational design was performed that yielded a second generation of six analogues (Gen2). Gen2 analogues were designed to investigate variations at the N-alkyl amino acid (Figures 3 and S8). Previously, it was reported that increasing the alkyl chain length in globomycin resulted in greater antimicrobial activity.^16^ Therefore, eight and twelve carbon atom chains were examined, along with polyethylene glycol (PEG) chains. Octyl derivatives of **G1a** and **G1b** were synthesized (**G2a** and **G2b**, respectively) as well as the corresponding N-alkyl glycine analogue (**G2c**), of which the dodecyl derivative was also synthesized (**G2d**). PEG analogues of this glycine modification were prepared with three and eight ethylene glycol units (**G2e** and **G2f**, respectively, Figure S8). Compared to the best Gen1 compounds, **G2e** showed reduced potency, with an IC_50_ value of 6.16 ± 0.84 μM. No inhibition was detected with **G2f** (Figure S11). For the alkyl chain analogues, **G2a** and **G2d** showed the most potent inhibition with IC_50_ values of 304 ± 62 and 157 ± 25 nM, respectively (Figures 3, S12, and S13). **G2b**, the R epimer analogue of **G2a**, showed a higher IC_50_ value of 430 ± 50 nM, while the shorter chain analogue **G2c** had lower potency (IC_50_ 920 ± 70 nM), although this still represents an improvement on the parent compound (Figure S11). A longer hydrocarbon chain provided an improved potency. Increasing hydrophobicity by using an Ala residue in place of Gly also enhanced potency, although necessitating correct stereochemistry. The most potent analogues, **G2a** and **G2d**, both displayed nanomolar IC_50_’s with the potential for further development as antibiotics.

**Figure 3:**
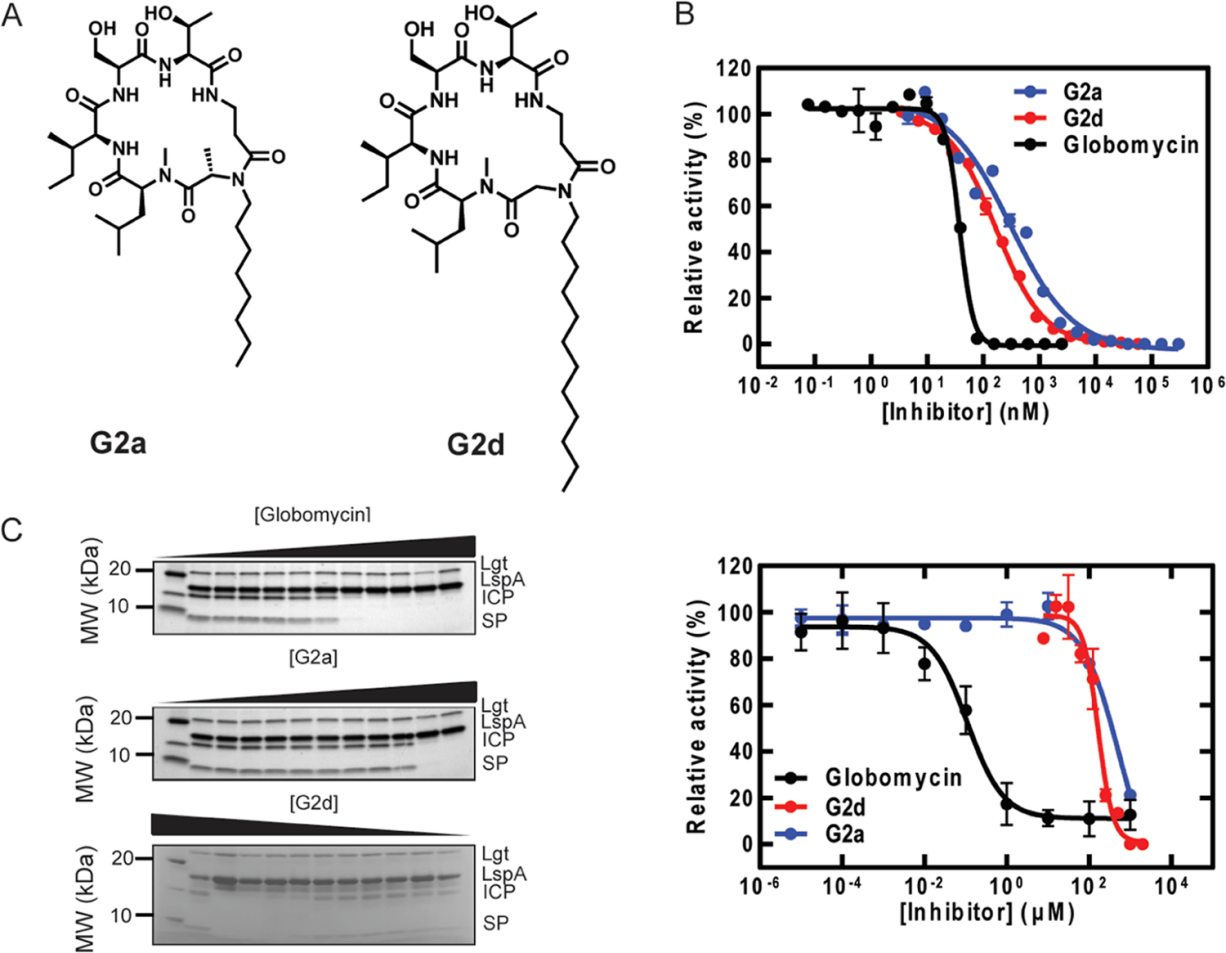
Globomycin analogues inhibit LspA activity *in vitro*. (A) Structures of **G2a** and **G2d**. (B) Results of the FRET dose–response assays. *Pa*LspA concentration was 40 nM, FRET substrate concentration was 50 μM, and the concentration of inhibitors globomycin, G2a, and G2d ranged from 0–3,000 nM. (C) SDS-PAGE gels and quantitation of the *Pa*LspA gel-shift dose–response assays. ppICP concentration was 12 μM, DOPG concentration was 600 μM, and Lgt concentration was 1.2 μM. The Lgt-catalyzed reaction was allowed to proceed for 60 min at 37 °C, after which inhibitors (0–1000 nM) were added. The *Pa*LspA reaction was initiated by the addition of 100 nM enzyme. The LspA reaction was allowed to proceed for 30 min at 37 °C before being quenched with SDS-PAGE loading buffer (62.5 mM Tris/HCl pH 6.8, 2.5% (w/v) SDS, 0.002% (w/v) bromophenol blue, 0.5 M β-mercaptoethanol, 10% (v/v) glycerol). Quantitation of the gel-shift assays was performed using Image Lab, and data were plotted using GraphPad Prism.

To validate the more potent analogues, **G2a** and **G2d** (Figure 3), as specific inhibitors of LspA (*Pa*LspA and *Ec*LspA), an orthogonal SDS–polyacrylamide gel electrophoresis (SDS-PAGE) gel-shift assay was used.^15^ In this assay, recombinant prepro inhibitor of cysteine protease (ppICP), representing the ppBLP, was first converted by Lgt to pICP using dioleoylphosphatidylglycerol (DOPG) as the lipid substrate. LspA then cleaved the SP from pICP, producing DA-ICP, resulting in a ∼10 kDa molecular weight shift that can be tracked by SDS-PAGE.^15^ Inhibition of LspA activity can be quantified by measuring the signal intensity of the product DA-ICP. This assay confirmed that the designed compounds **G2a** and **G2d** are specific inhibitors of LspA.

With these *in vitro* results, we sought to investigate the ability of **G2a**, **G2d**, and Gen1 compound **G1b** to inhibit the growth of reference and multi-drug-resistant bacteria, including *P. aeruginosa*, *Acinetobacter baumannii* and *Escherichia coli* (Table 1).

**Table 1:** MICs for the Designed Compounds **G1b**, **G2a**, and **G2d**

	MIC (μg/mL)				
	**globomycin**	**W1J**	**G1b**	**G2a**	**G2d**
*P. aeruginosa***950**	**16**	32	32	32	32
*E. coli***ATCC25922**	**10**	32	32	32	32
*A. baumannii***AB5075**	32	20	25	**16**	**16**
*A. baumannii***AB17978**	16	16	16	**12.5**	16

Minimum inhibitory concentration (MIC) values were measured for all three analogues and compared with MICs for globomycin and **W1J** (IC_50_ of 0.099 μM), an inhibitor identified by screening 646,275 potential small-molecule inhibitors, followed by SAR optimization.^19^ In the case of *P. aeruginosa*, globomycin showed the lowest MIC value at 16 μg/mL, while all other analogues gave values of 32 μg/mL. Likewise, for *E. coli*, globomycin gave an MIC of 10 μg/mL, with all other analogues having a higher value of 32 μg/mL. However, for both *A. baumannii* strains, the **G2a** and **G2d** analogues gave the lowest MICs of all compounds tested, at 16 μg/mL for *A. baumannii* AB5075 and 12.5 for *A. baumannii* AB17978. Similarly, Gen1 analogue **G1b** performed better than or comparable to globomycin against *Acinetobacter* strains. This level of antimicrobial activity compared to that recorded in vitro presumably arises from the improved stability of the rationally designed peptide linkages in the novel compounds. These results show that **G2a**, **G2d**, and **G1b** are effective and, therefore, are promising compounds for the development of antibiotics targeting Gram-negative pathogens. Importantly, some are more potent inhibitors of growth than globomycin against certain strains.

We have used computational peptide design to generate cyclic peptide analogues of the antibiotic globomycin. In contrast to rational design methods, biologically active analogues with IC_50_ values in the single-digit μM range were generated in the first round of designs using the *de novo* method. The second round produced more potent inhibitors with IC_50_ values in the high nM range. This approximately 10-fold increase in potency was achieved over just two generations, requiring the synthesis of only 12 compounds. The computational design approach enabled replacement of the labile, therapeutically limiting ester moiety in globomycin with a more stable amide. The most potent analogues, **G2a** and **G2d**, inhibited LspA *in vitro*, as revealed by both FRET and gel-shift assays. More importantly, both analogues showed antimicrobial activities comparable to or better than those of globomycin in microdilution assays against ESKAPE-E pathogens *E. coli*, *P. aeruginosa*, and two strains of *A. baumannii*. **G2a** and **G2d** exhibited lower MIC values against both *A. baumannii* strains compared to globomycin. Our computation-based strategy should enable targeting of other related lipoprotein peptidases with peptide macrocycles. *De novo* peptide design facilitates rapid access to biologically active lead candidates for therapeutic development, greatly accelerating the race against antibiotic resistance while alleviating the requirement for costly synthesis and screening approaches. We anticipate using this technology to develop more potent inhibitors of LspA and novel inhibitors of other bacterial targets to combat multi-drug-resistant Gram-negative bacteria.

## Data Availability

The data underlying this study are available in the published article and its Supporting Information.

## Supporting Information Available

Characterization data, experimental details, methods, and additional data (PDF)

## Abbreviations

BLP: bacterial lipoprotein
DA-BLP: diacylated BLP
DOPG: dioleoylphosphatidylglycerol
FRET: Förster resonance energy transfer
Lgt: lipoprotein diacylglyceryl transferase
Lnt: lipoprotein *N*-acytransferase
LspA: lipoprotein signal peptidase II
MH: membrane helix
MIC: minimum inhibitory concentration
pBLP: Pro-BLP
PEG: polyethylene glycol
PH: periplasmic helix
ppBLP: pre-proBLP
SAR: structure–activity relationship
SDS-PAGE: sodium dodecyl sulfate–polyacrylamide gel electrophoresis
SP: signal peptide
