# Supplementary material for: Computational Design of Cyclic Peptide Inhibitors of a Bacterial Membrane Lipoprotein Peptidase
Source: ACS Chem Biol. 2024 May 7;19(5):1125–30. doi: 10.1021/acschembio.4c00076 (PMC11106742; doi:10.1021/acschembio.4c00076)

Supplemental Information

Computational design of cyclic peptide inhibitors of a bacterial membrane lipoprotein peptidase

Timothy W. Craven,‡[1,2] Mark D. Nolan,‡[3] Jonathan Bailey,‡[4,5] Samir Olatunji,[4] Samantha J. Bann,[6] Katherine Bowen,[3] Nikita Ostrovitsa,[3] Thaina M. Da Costa,[7] Ross D. Ballantine,[6] Dietmar Weichert,[4] Paul M. Levine,[1,2] Lance J. Stewart,[1,2] Gaurav Bhardwaj,[1,2] Joan A. Geoghegan,[7,8,]* Stephen A. Cochrane,[6,]* Eoin M. Scanlan,[3,]* Martin Caffrey,[4,]* David Baker.[1,2,9,]*

[1] Department of Biochemistry, University of Washington, Seattle, WA 98195, USA.

[2] Institute for Protein Design, University of Washington, Seattle, WA 98195, USA.

[3] School of Chemistry, Trinity College Dublin, Dublin, D02 R590, Ireland.

[4] School of Medicine and School of Biochemistry and Immunology, Trinity College Dublin, Dublin, D02 R590.

[5] Biological Inorganic Chemistry Laboratory, The Francis Crick Institute, London NW1 1AT, UK.

[6] School of Chemistry and Chemical Engineering, Queen's University Belfast, David Keir Building, Stranmillis Road, Belfast, UK, BT9 5AG.

[7] Department of Microbiology, Moyne Institute of Preventive Medicine, School of Genetics and Microbiology, Trinity College Dublin, D02 VF25, Ireland.

[8] Institute of Microbiology and Infection, College of Medical and Dental Sciences, University of Birmingham, Birmingham B15 2TT, UK.

[9] Howard Hughes Medical Institute, University of Washington, Seattle, WA 98195, USA.

**Table of Contents:**	**Page**
**Materials and Methods.**	**S3**
**Figure S1.** Rationally designed globomycin peptide analogues with no activity against LspA from *P. aeruginosa*.	**S6**
**Figure S2.** Solid-phase peptide synthesis of cyclic globomycin N-L-lysine amide isosteres, acylated with linear lipids, ranging from C3 – C8 in carbon chain length.	**S7**
**Figure S3.** Solid-phase peptide synthesis of analogues containing an 8-C lipid chain with variation in ring-size and stereochemistry at the lipid-bearing amide.	**S7**
**Figure S4.** Solid-phase peptide synthesis of cyclic octyl-globomycin dicarba isostere.	**S8**
**Table S1.** Analytical and MIC (*E. coli*) data for synthetic analogues.	**S9**
**Supplementary discussion for rationally designed compounds.**	**S10**
**Figure S5.** Generation 1 globomycin peptide analogues that inhibit *Pa*LspA activity.	**S11**
**Figure S6.** Dose-response and Morrison plots of *Pa*Lspa inhibition by the Generation 1 R-enantiomer compounds.	**S13**

**Figure S7.** Dose-response and Morrison plots of *Pa*LspA inhibition by the Generation 1 S-enantiomer compounds.	**S14**
**Figure S8.** Generation 2 globomycin peptide analogues that inhibit *Pa*LspA activity with lower potency than compounds G2a and G2d.	**S15**
**Figure S9.** HPLC and MS analysis of the compound G2a.	**S16**
**Figure S10.** HPLC and MS analysis of the compound G2d.	**S17**
**Figure S11.** Dose-response and Morrison plots of *Pa*LspA inhibition by the less potent Generation 2 compounds.	**S18**
**Figure S12.** Dose-response and Morrison plots of PaLspA inhibition by compound G2d.	**S19**
**Figure S13.** Dose-response and Morrison plots of *Pa*LspA inhibition by compound G2a.	**S20**
**Figure S14.** *Pa*LspA and *Ec*LspA gel-shift inhibition assays by globomycin, G2a and G2d.	**S21**
**References**	**S22**
**MS Spectra for Generation 1 Peptides**	**S23**
**MS Spectra for Generation 2 Peptides**	**S29**

Materials & Methods

Materials: All reagents were obtained from commercial sources and used without additional purification. All aqueous solutions were prepared using ultrapure laboratory grade water (deionized, filtered, and sterilized) obtained from an in-house ELGA water purification system. Reverse-phase high-performance liquid chromatography (RP-HPLC) was performed using an Agilent Technologies 1260 Series HPLC instrument with a diode array detector. For analytical analysis, a C_{18} reversed-phase HPLC column was used (Higgins). Samples were eluted with a 5-95% acetonitrile/water gradient (0.1% TFA) in 45 min with a flow rate of 1 mL/min and monitored by absorbance at 214 nm. For purifications, semi-preparative C_{18} reversed-phase HPLC columns were used (Higgins). Samples were eluted with a 5-65% or 25-95% acetonitrile/water gradient (0.1% TFA) in 35 min with a flow rate of 5.0 mL/min, and monitored by absorbance at 220 nm. Analytical HPLC analysis of final compounds **G2a** and **G2d** was performed on a Shimadzu Nexera HPLC instrument equipped with a diode array detector and a C_8 reversed-phase analytical column. Mass spectra were acquired on an Agilent LC-TOF or Thermo ESI direct inject mass spectrometer.

Peptide Synthesis: Peptides were synthesized using standard Fmoc solid-phase chemistry on 2-Chlorotrityl ProTide (CEM, 0.45 mmol/g) resin using a Liberty Blue peptide synthesizer from CEM. All peptides were synthesized on a 0.1 mmol scale and yielded ~20-60% product. Couplings were performed using DIC (5 equiv, Novabiochem) and Oxyma (10 equiv, Sigma) in DMF followed by Fmoc deprotection with 20% piperidine. *N*-alkyl amino acids were installed by coupling of the appropriate α-bromo acid, followed by treatment with the appropriate amine (2 equiv.) in DMSO for 2 h. Peptides were cleaved (96.5:2.5:1 DCM/triisopropylsilane/TFA) for 1 h at room temperature (20 ºC) and precipitated from cold diethyl ether. Peptides were then resuspended in DCM and cyclized using COMU coupling agent, followed by deprotection (95:2.5:2.5 TFA/H2O/triisopropylsilane) for 3.5 h at room temperature. Cyclic peptides were then purified by reverse-phase preparative HPLC. All peptides were characterized by mass analysis using ESI-MS, and the sample purity was assessed by analytical HPLC.

Expression and purification of *Pa*LspA, Lgt, *Ec*LspA and ppICP: *Pa*LspA, Lgt and pre-proICP (ppICP) were expressed and purified using previously published protocols (Vogely *et al*, 2016).[1] *Ec*LspA was expressed and purified as described for *Pa*LspA, with the following modification. The lysis buffer was 50 mM Hepes pH 7.5, 150 mM NaCl, 1 mM TCEP, 10 %(v/v) glycerol. Isolated membranes, containing *Ec*LspA, were solubilized with 1 %(v/v) FC12 for 2 h at 4 ºC. The final storage buffer (size-exclusion buffer) was 50 mM Hepes, 150 mM NaCl, 1 mM TCEP, 10 %(v/v) glycerol, 0.14 %(w/v) FC12.

FRET-based peptidase assay: The FRET-based assay is a continuous assay that monitors an increase in fluorescence at excitation and emission wavelengths of 320 and 420 nm respectively. The synthesis and purification of the FRET substrate has been described previously.[1] Inhibitor concentrations ranged from 0 to 600 µM. Assay buffer was 100 mM MES/NaOH pH 5.6, 150 mM NaCl and 0.05 %(w/v) LMNG. Final DMSO concentration in all reactions was 10 %(v/v). For *Pa*LspA, dose-response assays were performed using 500 nM (Generation 1 compounds) or 100 nM (Generation 2 compounds) and 50 µM FRET peptide substrate. The reaction mixtures were pipetted into wells of a 96- (Thermo Scientific) or a 384-well plate (4titude®) and incubated for 10 min at 37 °C in a SpectraMax M3 (Molecular devices), prior to starting the assay. Assays were

initiated by additing enzyme and were performed at 37 °C. Initial velocities (v_o and v_i) were determined from the linear region (steady-state) of the reaction progress curve. Initial velocity data was fitted to the dose-response inhibition equation (*Equation 1*) to determine IC_{50} values and to the Morrison equation (*Equation 2*) to determine *Ki'* using GraphPad Prism® (Vogeley *et al*, 2016; Olatunji *et al*, 2020).[1,2]

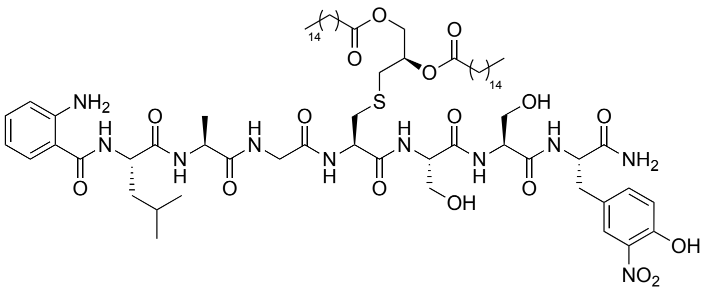

Structure of the FRET lipopeptide substrate used for assaying the peptidase activity of LspA.[2]

Gel shift assay: The gel-shift assay is a non-continuous SDS-PAGE based assay that monitors the formation of diacylglyceryl-ICP and signal peptide, products of the LspA catalysed reaction. Gel-shift assays were set up according to published protocols (Vogeley *et al*, 2016; Olatunji *et al*, 2020),[1,2] with the following modifications. 12 µM pre-proICP (ppICP) (Lgt substrate), 250 or 600 µM DOPG (Lgt substrate) (Avanti Polar Lipids, Inc.) and 1.2 µM Lgt in 50 mM Tris/HCl pH 7.5, 150 mM NaCl, 1 mM DTT, 0.02%(w/v) LMNG was mixed and incubated at 37 °C and 200 rpm for 60 min to allow for the Lgt catalyzed conversion of pre-proICP to the LspA substrate proICP (pICP). Inhibitors (globomycin, **G2a** or **G2d**) (0 – 1,000 µM final concentration) in DMSO were added to the reaction mix and the LspA reaction was initiated by the addition of 100 nM *Pa*LspA or *Ec*LspA. The LspA reaction was performed at 37 °C and allowed to proceed for 30 min, then 20 µL aliquots were removed and the reaction was stopped by adding 10 µL 4 x SDS loading buffer (62.5 mM Tris/HCl pH 6.8, 2.5 %(w/v) SDS, 0.002%(w/v) bromophenol blue, 0.5 M β-mercaptoethanol, 10%(v/v) glycerol). 10 µL of the stopped reaction mixtures were loaded on precast Mini-PROTEAN® gels (Bio-Rad) and run with Tris/glycine buffer (25 mM Tris/HCl pH 8.0, 250 mM glycine, 0.1 %(w/v) SDS). Gels were stained using InstantBlue™ (Expedeon) and imaged using a Bio-Rad Gel-Doc imager. LspA activity was determined by tracking the band intensity of the diacylglyceryl-ICP product which was quantified using Image Lab. IC_{50} values were determined using GraphPad Prism by fitting the data to *equation 1.*

Microdilution assay:

MIC values were determined in cation-adjusted Mueller-Hinton broth using the broth microdilution method according to CLSI guidelines. Plates were incubated for 16 to 24 h at 37 °C. Six biological replicates were performed across three different 2-fold dilution series, starting at concentration of 100 µg/ml, 80 µg/ml or 64 µg/ml. The lowest concentration of antimicrobial inhibiting growth was taken as the MIC. This was established by visual inspection of the medium after the incubation period with a lack of turbidity of the growth medium indicating no growth.[3]

Data analysis: All data was fitted using GraphPad Prism 9.0.0 as detailed in (Vogeley *et al*, 2016; Olatunji *et al*, 2020).[1,2]

Equation 1. Dose-response

Y=Bottom + (Top-Bottom)/(1+(IC50/X)^HillSlope)

Equation 2. Morrison equation
Y=Vo*(1-((((Et+X+(Ki*(1+(S/Km))))-(((Et+X+(Ki*(1+(S/Km))))^2)-4*Et*X)^0.5))/(2*Et)))

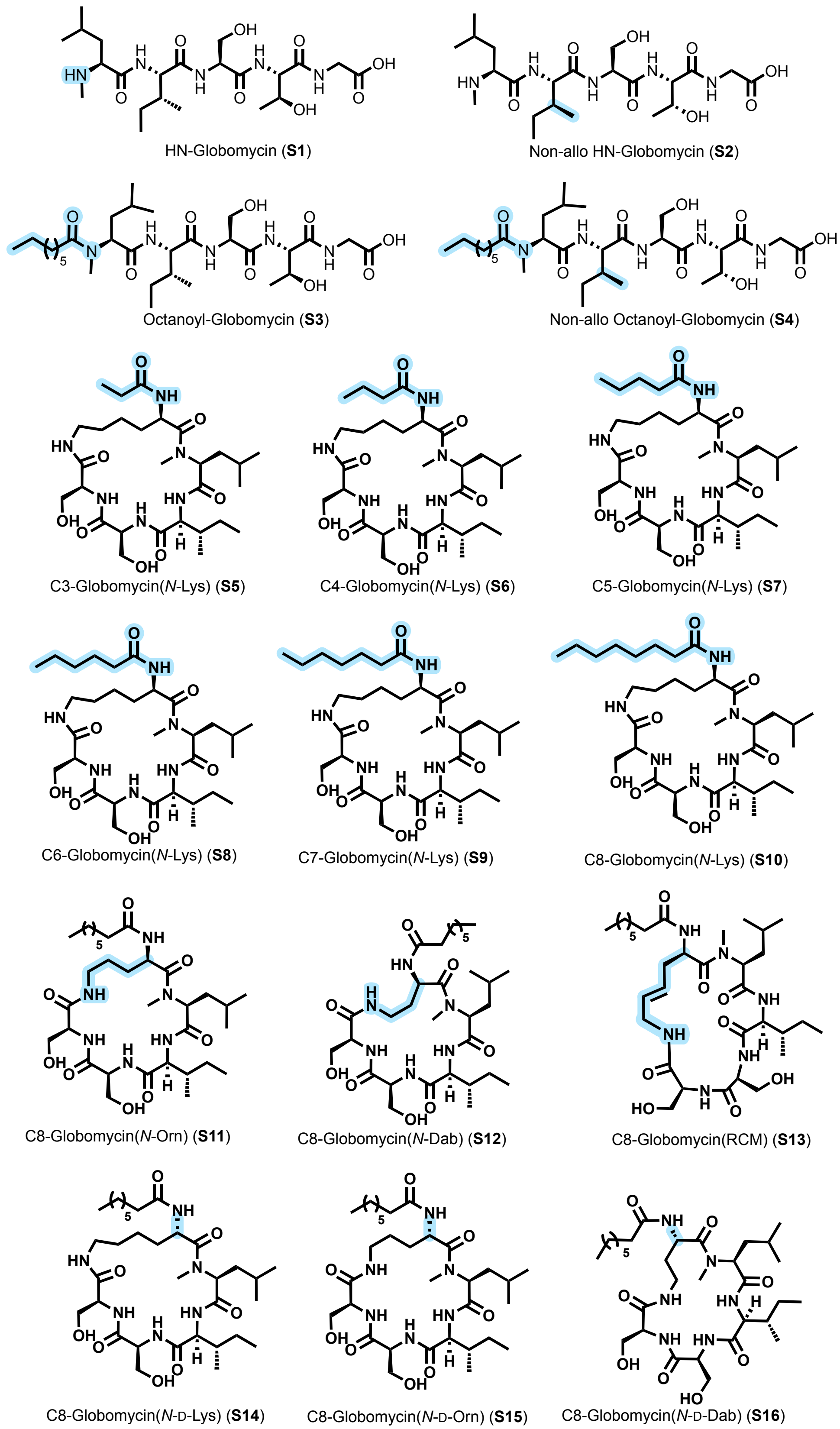

Figure S1. Rationally designed globomycin peptide analogues with no activity against *Pa*LspA. Blue shading indicates the areas of variation for each analogue.

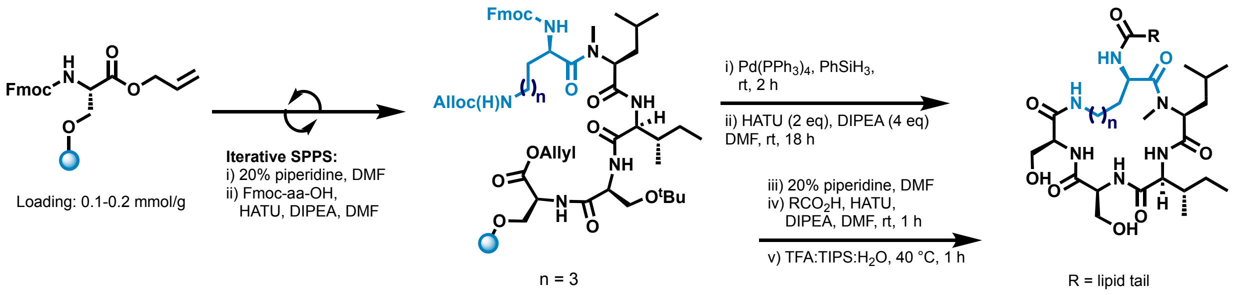

Figure S2. Solid-phase peptide synthesis of cyclic globomycin *N*-L-lysine amide isosteres with alkyl chains from 3 to 8 carbon atoms long. Peptides were synthesized on a 0.1 mmol scale, from an Fmoc-L-Ser(2-CTC)-OAll solid-support and cyclized on resin using HATU (2 eq) and DIPEA (4 eq) between the side-chain of an *N*-terminal L-lysine and *C*-terminal L-serine residue.

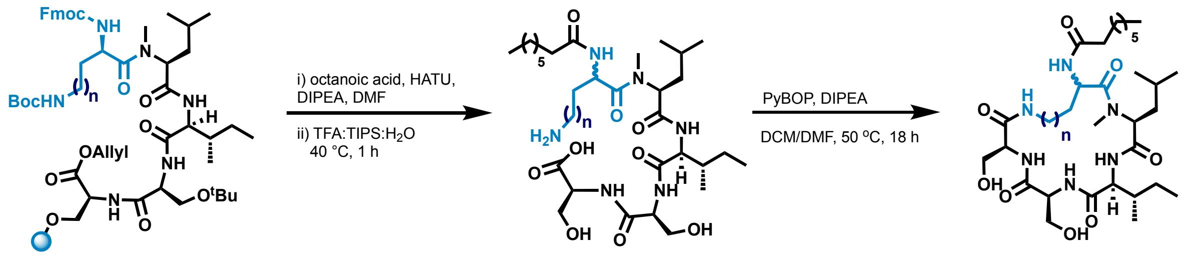

Figure S3. Solid-phase peptide synthesis of analogues containing an 8-C lipid chain with variation in ring-size and stereochemistry at the lipid-bearing amide. Peptides were synthesized on a 0.1 mmol scale, using side-chain immobilised Fmoc-L-Ser(2-CTC)-OAll solid-support (shaded sphere), to generate the side-chain immobilized common tetrapeptide intermediate. The L- or D-stereoisomer of cationic amino acids lysine, ornithine, or diaminobutyric acid was coupled at the *N*-terminal and cyclized in solution.

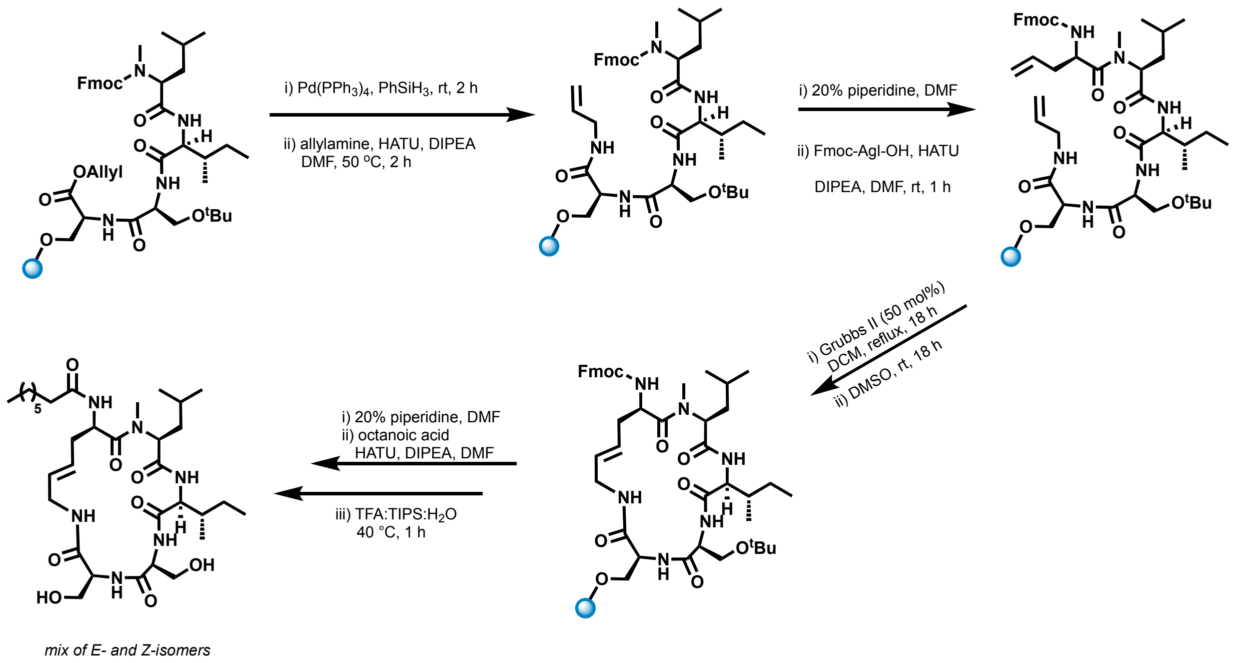

Figure S4. Solid-phase peptide synthesis of cyclic octyl-globomycin dicarba isostere. SPPS was carried out on a 0.07 mmol scale using side-chain immobilised Fmoc-L-Ser(2-CTC)-OAll solid-support to generate the side-chain immobilized linear diene precursor. Cyclization was achieved via an on-resin ring-closing metathesis (RCM) reaction employing Grubbs 2nd generation catalyst (50 mol%)

Table S1. Analytical and MIC (*E. coli*) data for synthetic analogues

	Compound	HRMS			MIC
		Formula	MS Calcd.	MS found	(µg/mL)
Linear Analogues	**S1**	$C_{30}H_{57}N_5O_8$	616.4280	616.4286	>200
	**S2**	$C_{30}H_{57}N_5O_8$	616.4280	616.4286	>200
	**S3**	$C_{30}H_{55}N_5O_9$	377.2471	377.2032	>200
	**S4**	$C_{30}H_{55}N_5O_9$	377.2471	377.2033	>200
Lipid variants	**S5**	$C_{28}H_{50}N_6O_8K$	637.3328	637.3312	>200
	**S6**	$C_{29}H_{51}N_6O_8$	611.3767	611.3776	>200
	**S7**	$C_{32}H_{54}N_6O_{10}F_3$	739.3859	719.3865	>200
	**S8**	$C_{31}H_{55}N_6O_8$	639.4082	639.4106	>200
	**S9**	$C_{32}H_{58}N_6O_8Na$	677.4215	677.4209	100
	**S10**	$C_{33}H_{60}N_6O_8Na$	691.4371	691.4340	>200
Varied macrocycle size	**S11**	$C_{32}H_{57}N_6O_8$	653.4239	653.4340	>200
	**S12**	$C_{31}H_{55}N_6O_8$	639.4082	639.4114	>200
	**S13**	$C_{33}H_{58}N_6O_8Na$	689.4214	689.4214	>200
Varied stereochem.	**S14**	$C_{33}H_{59}N_6O_8$	667.4396	667.4423	>200
	**S15**	$C_{32}H_{57}N_6O_8$	653.4239	653.4253	>200
	**S16**	$C_{31}H_{57}N_6O_8$	641.4239	641.4243	>200

Supplementary discussion for rationally designed analogues.

Computational Methods

Our computational analysis commenced with the initial crystal structure of globomycin bound to the LspA protein, as delineated in Vogley et al.[1] This foundational structure served as our starting point for exploring the potential of various backbone chemistries to mimic or enhance the interaction between globomycin and LspA.

To systematically evaluate the impact of different backbone modifications on the structure and binding efficiency of globomycin, we employed a structure prediction framework as outlined in previously established methodologies.[4,5] This approach allowed us to simulate and analyze a wide array of cyclic peptide designs, each incorporating different backbone chemistries, while rigorously predicting their conformations.

A key criterion for assessing the viability of each designed peptide was its ability to preserve the critical binding residues of globomycin: N-Methyl-L-Leucine, allo-Isoleucine, L-Serine, and allo-L-Threonine. These residues, crucial for the binding efficacy of globomycin as observed in the PDB ID 5DIR, were meticulously maintained across all computational models to ensure the integrity of the interaction with LspA.

Through this computational process, peptides that exhibited predicted structures closely aligning with the essential globomycin binding conformation were identified. The primary focus was on cyclic peptides whose predicted states not only replicated the necessary binding residues but also demonstrated a predominant conformational state, indicating a high likelihood of successful binding in vivo.

In a bid to overcome *in vivo* hydrolysis of globomycin by esterase enzymes, the native ester linkage was replaced by an amide or dicarba linkage. To this end, a panel of twelve cyclic analogues were synthesized in which the native L-*allo*-threonine residue at position 4 was replaced by L-serine to aid side-chain resin loading onto 2-chlorotrityl resin and thus increase the rate of synthesis output. Fortunately, structure-activity relationship studies carried out by Kiho *et al.* confirm that position 4 can tolerate modifications at the side-chain of L-*allo*-threonine without detrimentally impacting antimicrobial activity.[3] Owing to side-chain resin immobilization, SPPS could proceed in both the *N*- and *C*-directions and so facilitate an on-resin cyclization of both amide and dicarba isosteres.

Initially, a library of six lipid amide analogues was synthesized via cyclization between the side-chain epsilon -NH_2 of an *N*-terminal L-lysine residue and the C-terminus of an L-serine residue. Each analogue was acylated with a simple linear lipid, ranging from C3 to C8 in length (**Figure S1**). To further probe the potential of the amide isostere, five additional analogues were synthesized in which the D- or L-stereoisomer of cationic amino acids diaminobutyric acid (Dab), ornithine (Orn), or Lys were used as the *N*-terminal cyclization partner. Each analogue was acylated with an octanoyl tail and the subsequent effects of ring size and/or stereochemistry, as well as lipid length, determined by assessing antimicrobial activity against *E. coli*. Peptides were also screened against *S. aureus.* However, these results have been omitted as globomycin is inherently inactive against Gram-positive strains.[4] Unfortunately, each analogue was unable to

inhibit bacterial growth, with MIC values of 100 to 200 mg/mL registered against *E. coli* (**Table S1**).

Additionally, C3 – C5 (peptides **S5** – **S7**) and C7-Gbm(NLys) (peptide **S9**) amide analogues were submitted for enzyme inhibition assays to determine if these cyclic amide isosteres could still inhibit the target enzyme, LspA. Each peptide was incubated with *Pa*LspA and the observed reduction in enzyme activity reported. Unfortunately, no reduction in activity was observed with peptides **S6** and **S7** (C4 and C5-Gbm(NLys) and only a moderate decrease in enzyme activity was found for peptides **S5** and **S9** (C3 and C7-Gbm(NLys).

An olefin isostere was also investigated as a potential replacement for the native ester linkage in globomycin. Octyl-globomycin(RCM) (isostere **S13**) was synthesized via side-chain immobilization of L-Ser4 and an on-resin ring-closing metathesis (RCM) reaction performed with Grubbs 2nd generation catalyst to generate a mix of *cis*- and *trans*-isomers. Analogously, peptide **S13** (RCM peptide) was void of antimicrobial activity against Gram-negative bacteria. These findings highlight the difficulty of successfully replacing the ester linkages in globomycin while retaining antimicrobial activity and native mechanism of action.

Four linear globomycin analogues were synthesized to determine the necessity of the cyclic core. The pentapeptide core of NMeLeu-Ile-Ser-Thr-Gly-OH was synthesized as both the natural *allo*-version and the cheaper non-*allo* version in which native residues *allo*-L-isoleucine and *allo*-L-threonine were replaced by their cheaper non-*allo* derivatives. Each linear pentapeptide was synthesized in both its acylated, octyl-derivative and non-acylated form and submitted for analysis of its *Pa*LspA inhibitory activity. No *Pa*LspA inhibition was observed, confirming the importance of globomycin's cyclic core for antimicrobial activity.

G1a

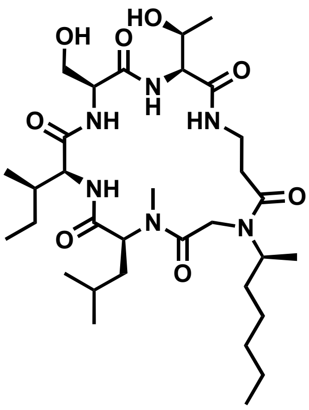

G1b

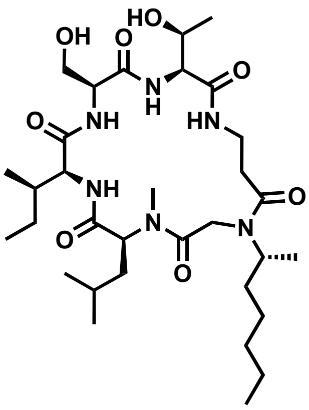

G1c

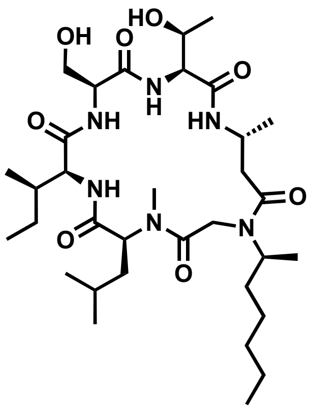

G1d

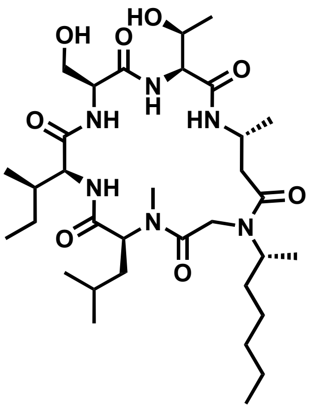

G1e

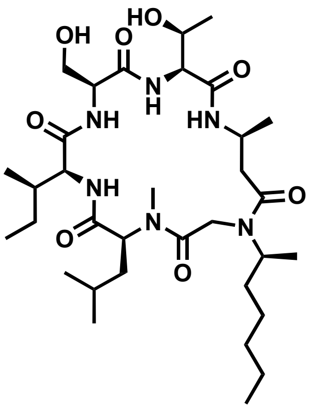

G1f

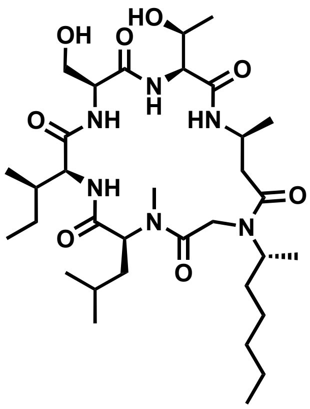

Figure S5. Generation 1 globomycin peptide analogues that inhibit *Pa*LspA activity.

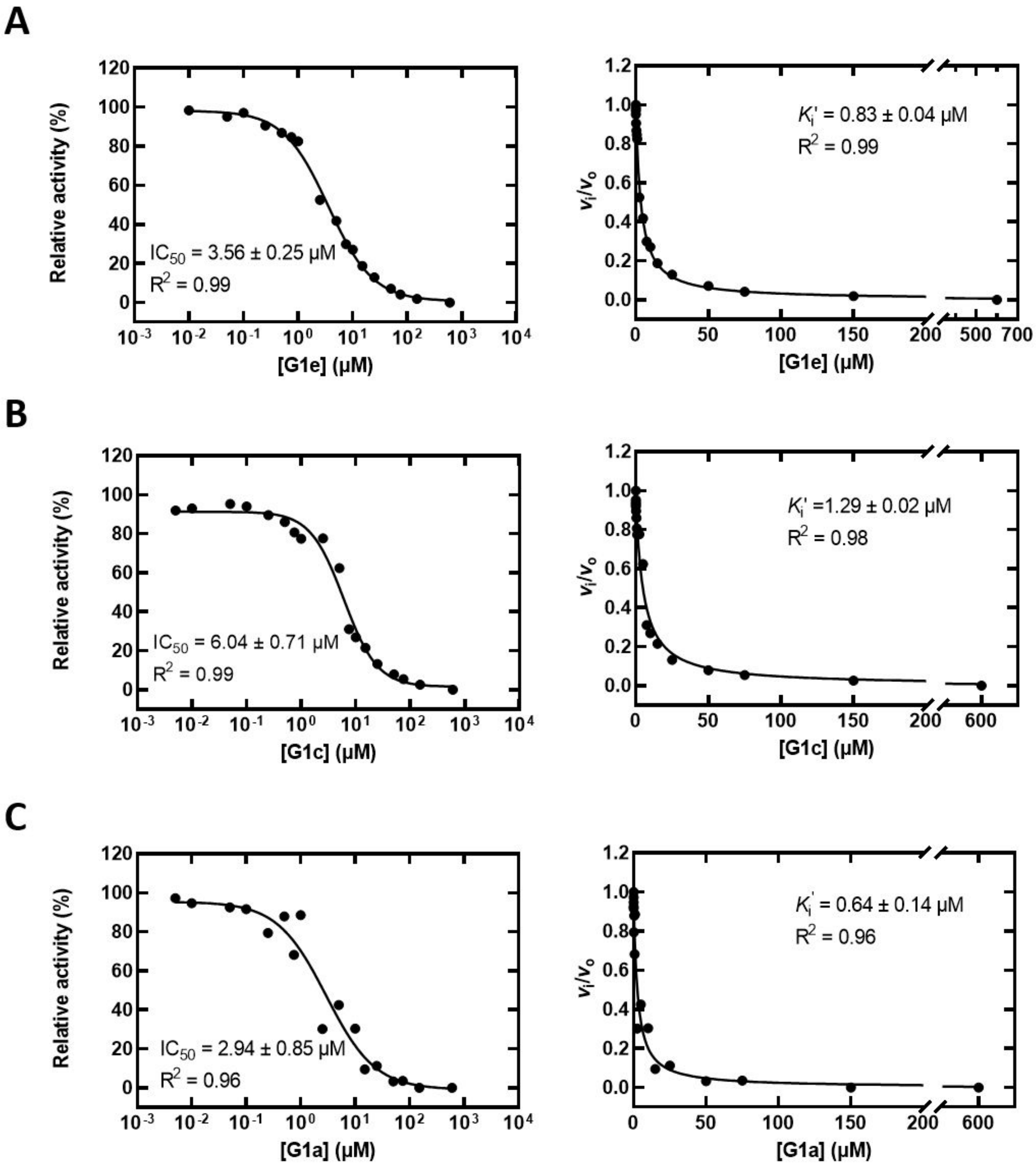

Figure S6. Dose-response and Morrison plots of *Pa*LspA inhibition by the Generation 1 R-enantiomer compounds. FRET-based peptidase assay. (**A**) Compound G1e. (**B**) Compound G1c. (**C**) Compound G1a. *Pa*LspA concentration was 500 nM, FRET substrate concentration was 50 μM and inhibitor concentrations ranged from 0 to 600 μM. Assays were run at 37 ^{o}C.

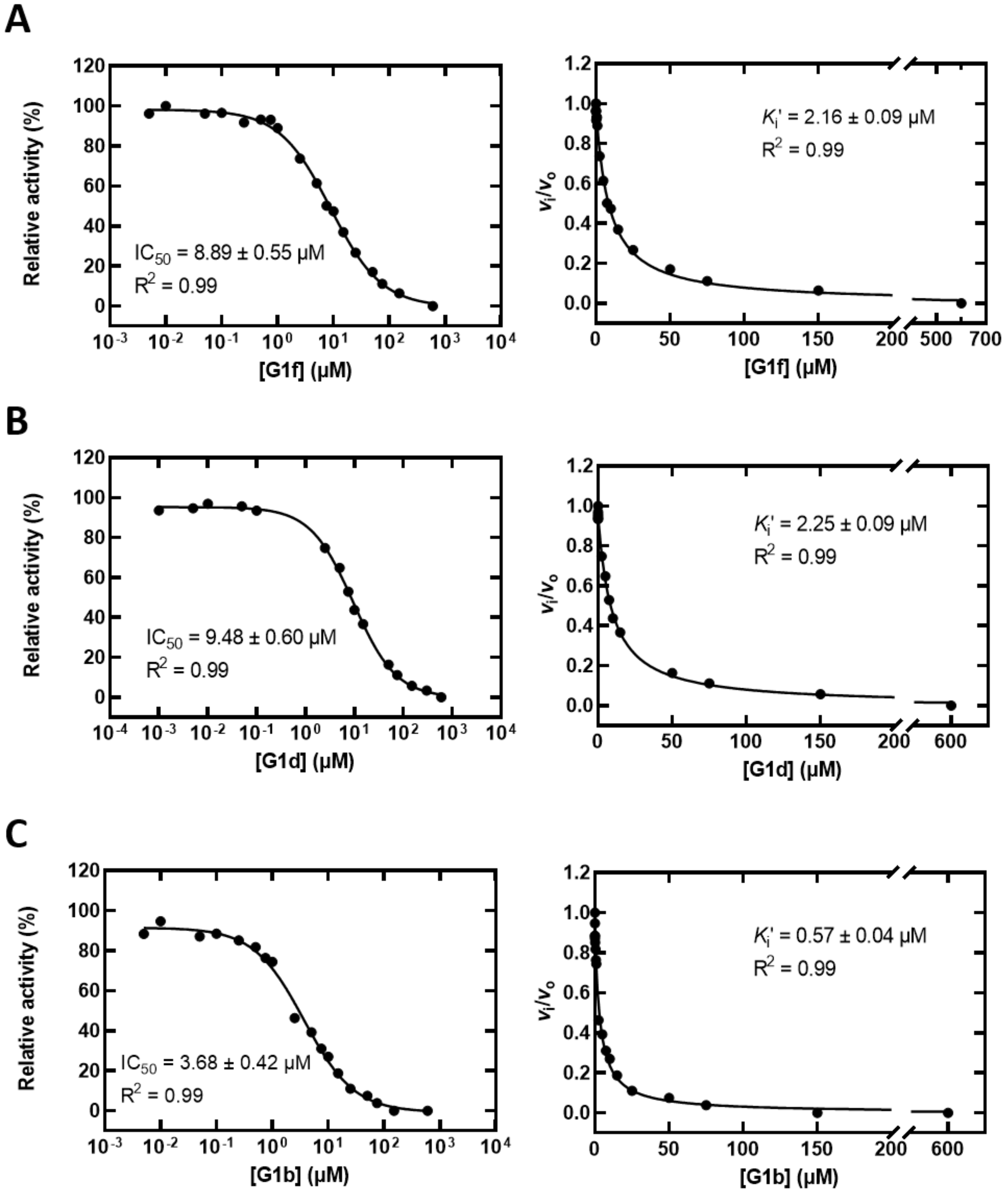

Figure S7. Dose-response and Morrison plots of *Pa*LspA inhibition by the Generation 1 S-enantiomer compounds. FRET-based peptidase assay. (**A**) Compound G1f. (**B**) Compound G1d. (**C**) Compound G1b. *Pa*LspA concentration was 500 nM, FRET substrate concentration was 50 μM and inhibitor concentrations ranged from 0 to 600 μM. Assays were run at 37 ^{o}C.

G2b

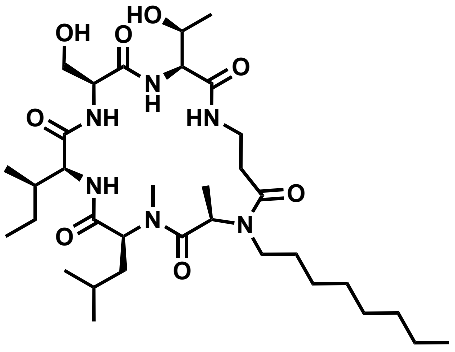

G2c

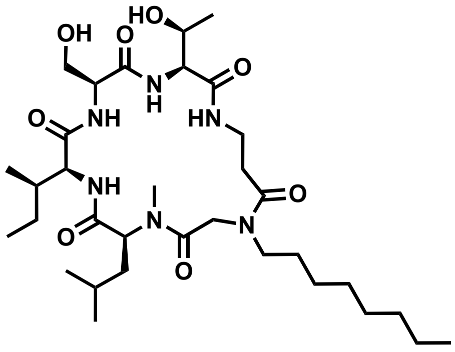

G2e

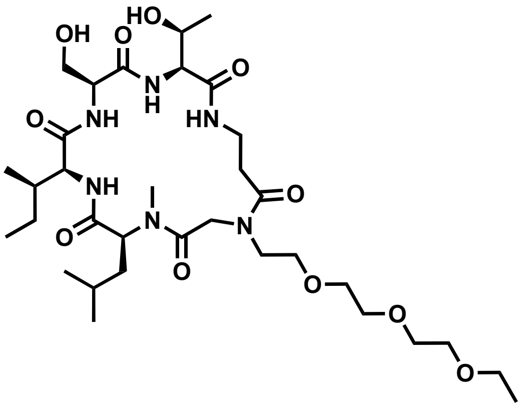

G2f

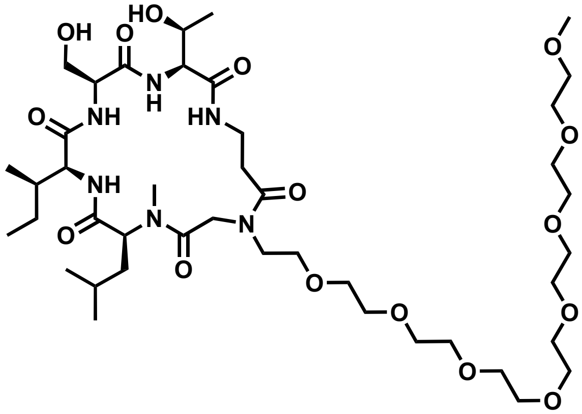

Figure S8. Generation 2 globomycin peptide analogues that inhibit LspA activity with lower potency than compounds G2a and G2d.

(A)

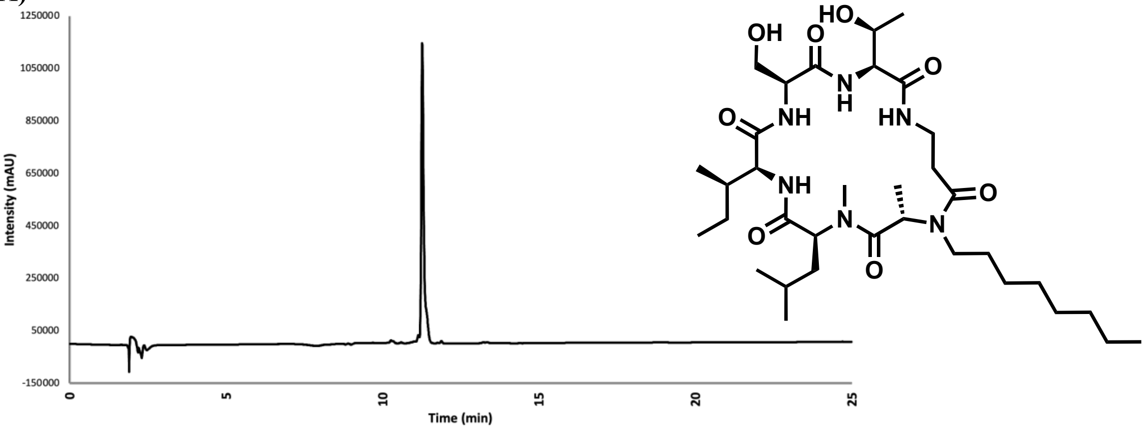

(B)

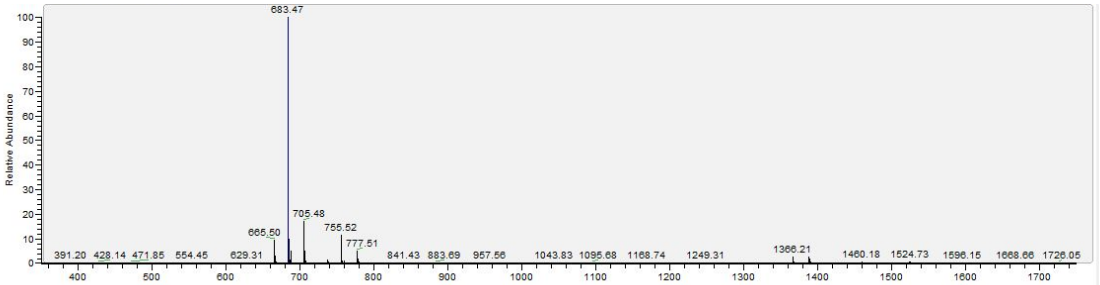

Figure S9. HPLC and MS analysis of compound G2a. (A) HPLC method: 1.0 mL/min, 5-95% ACN from 1-6 min, 95% ACN from 6-21 min. C8 stationary phase. (**B**) MS: [M+H]+ calculated 683.47 Da, found 683.47 Da.

(A)

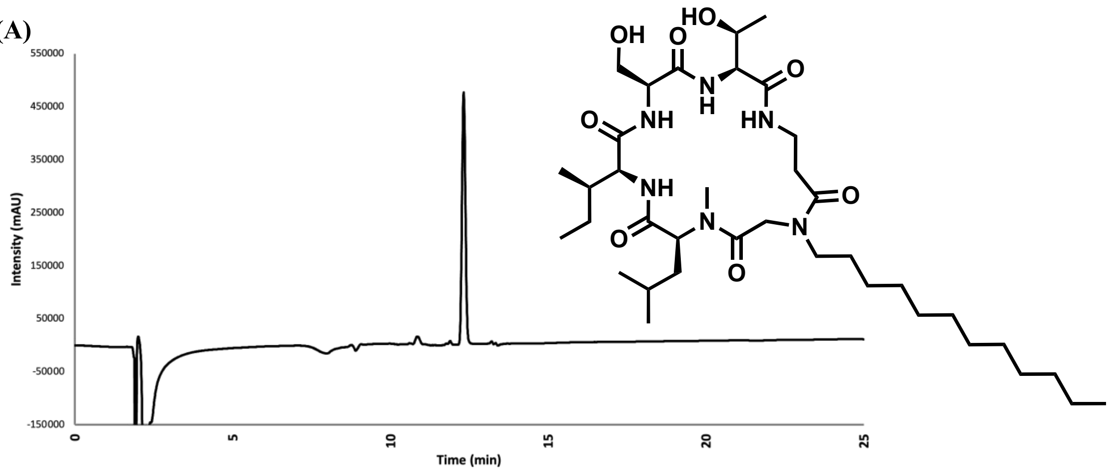

(B)

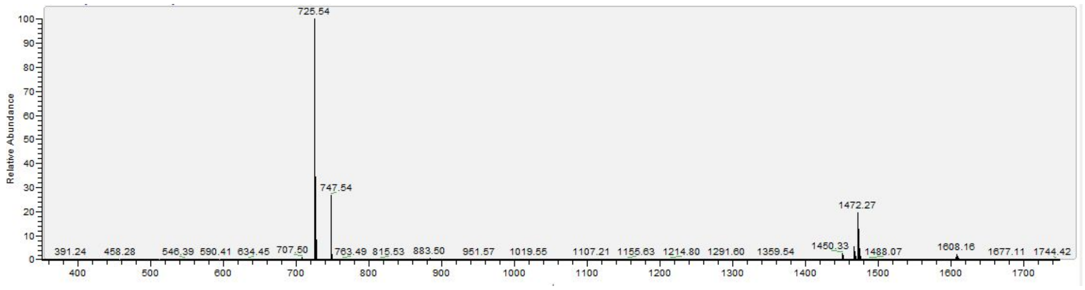

Figure S10. HPLC and MS analysis of the compound G2d. (A) HPLC method: 1.0 mL/min, 5-95% ACN from 1-6 min, 95% ACN from 6-21 min. C8 stationary phase. **(B)** MS: [M+H]+ calculated 725.54 Da, found 725.52 Da.

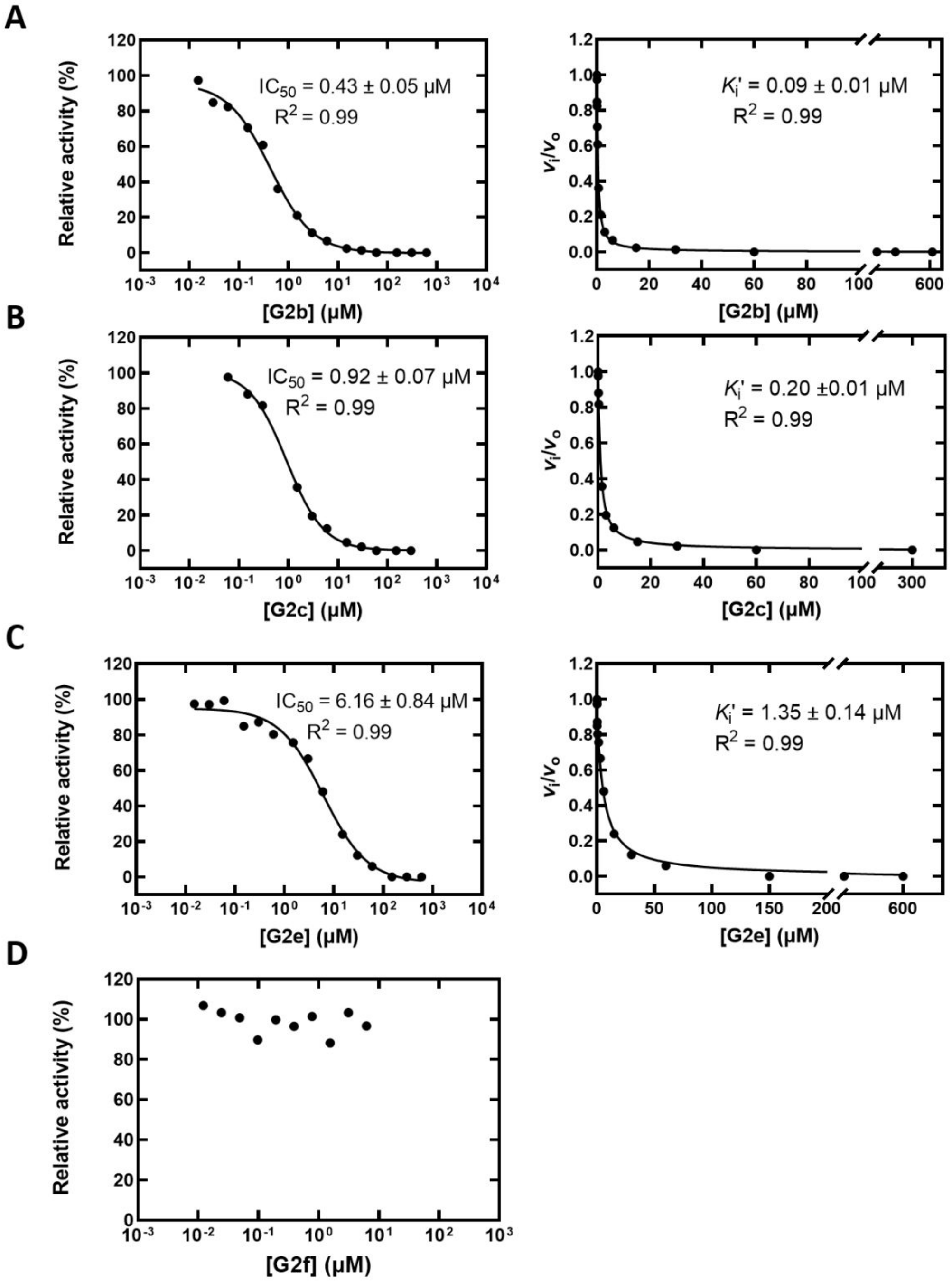

Figure S11. Dose-response and Morrison plots of *Pa*LspA inhibition by the less potent Generation 2 compounds. FRET-based peptidase assay. (**A**) Compound G2b. (**B**) Compound G2c (**C**) Compound G2e. (**D**) Compound G2f. *Pa*LspA concentration was 100 nM, FRET substrate concentration was 50 µM and inhibitor concentrations were 0 to 600 µM. Assays were run at 37 ^{o}C.

A

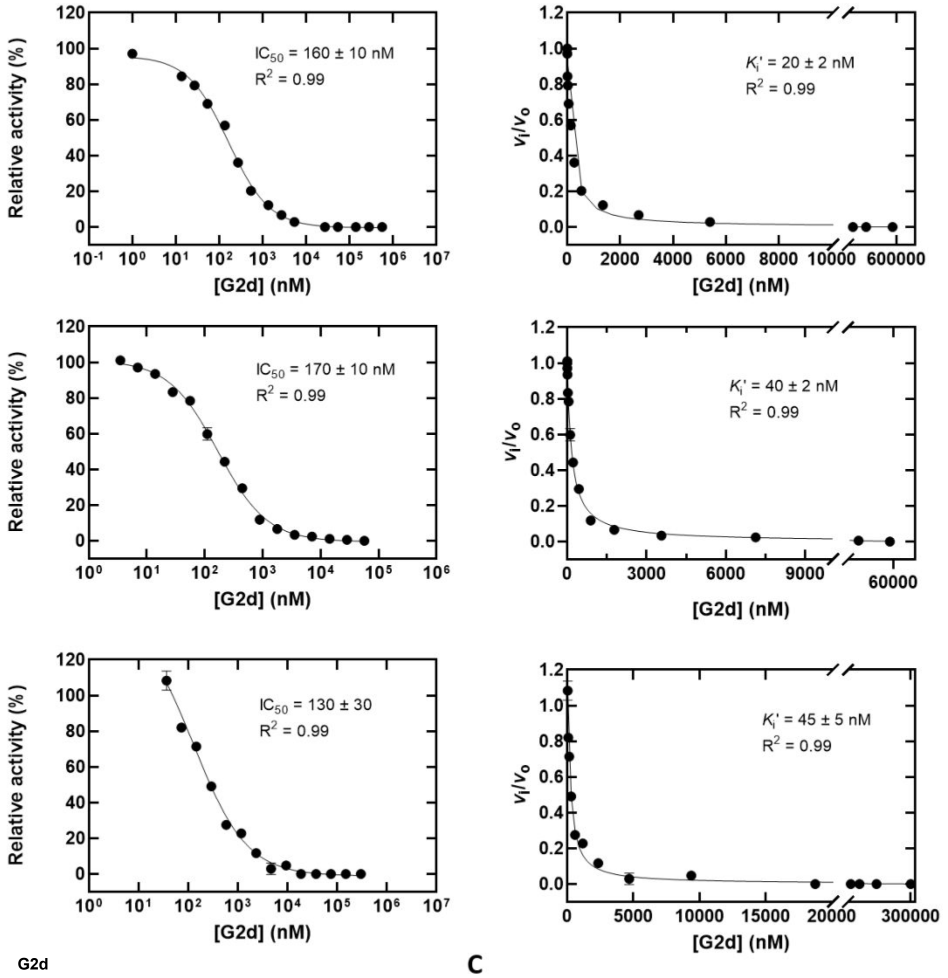

B

G2d

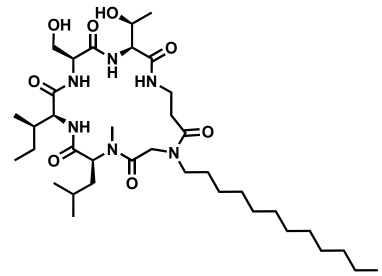

C

**IC50**	**150 ± 30 nM**
***K*i'**	**35 ± 15 nM**

Values are the average of 3 independent experiments and error is the standard deviation

Figure S12. Dose-response and Morrison plots of *Pa*LspA inhibition by compound G2d. FRET-based peptidase assays **(A)** Dose-response and Morrison plots. (**B**) Structure of compound G2d. (**C**) Average IC_{50} and K_i' values from the data shown in (A). *Pa*LspA concentration was 40 or 100 nM, FRET substrate concentration was 50 µM and inhibitor concentrations ranged from 0 to 600 µM. Assays were run at 37 °C.

A

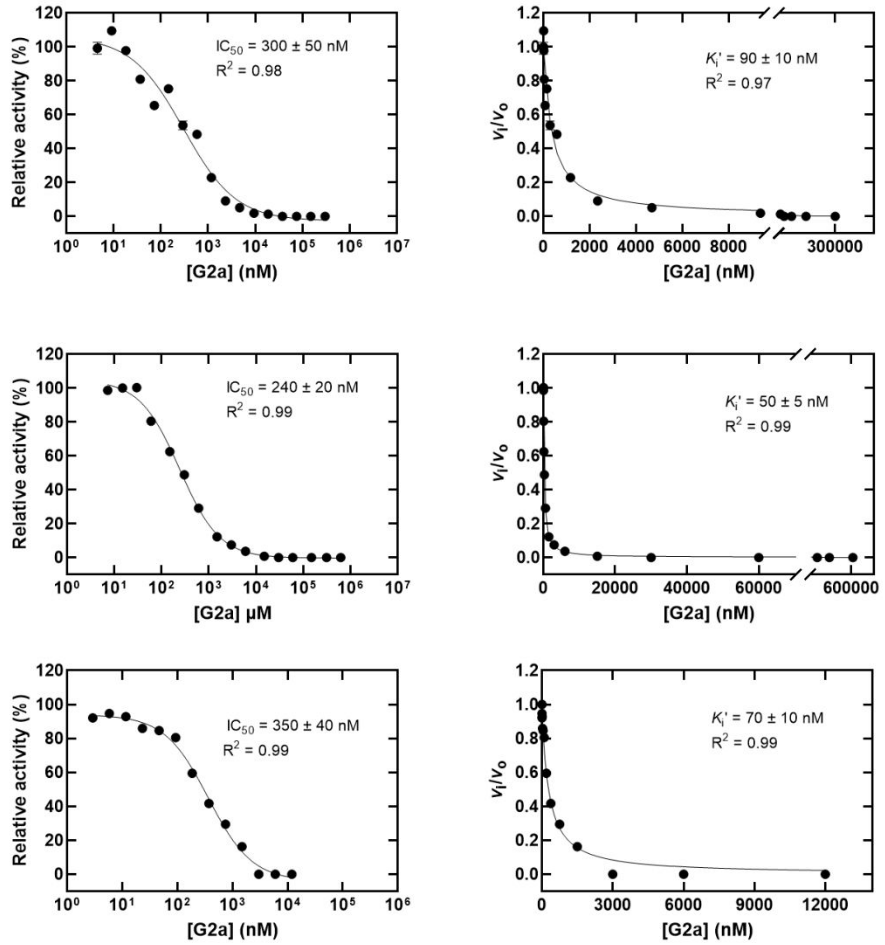

B G2a

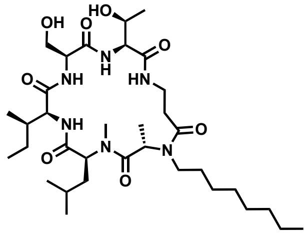

C

IC_{50}	300 ± 50 nM
K_i’	70 ± 20 nM

Values are the average of 3 independent experiments and error is the standard deviation

Figure S13. Dose-response and Morrison plots of *Pa*LspA inhibition by compound G2a. FRET-based peptidase assays **(A)** Dose-response and Morrison plots. (**B**) Structure of compound G2a. (**C**) Average IC_{50} and K_i’ values from the data shown in (A). *Pa*LspA concentration was 40 or 100 nM, FRET substrate concentration was 50 µM and inhibitor concentrations ranged from 0 to 600 µM. Assays were run at 37 ^oC.

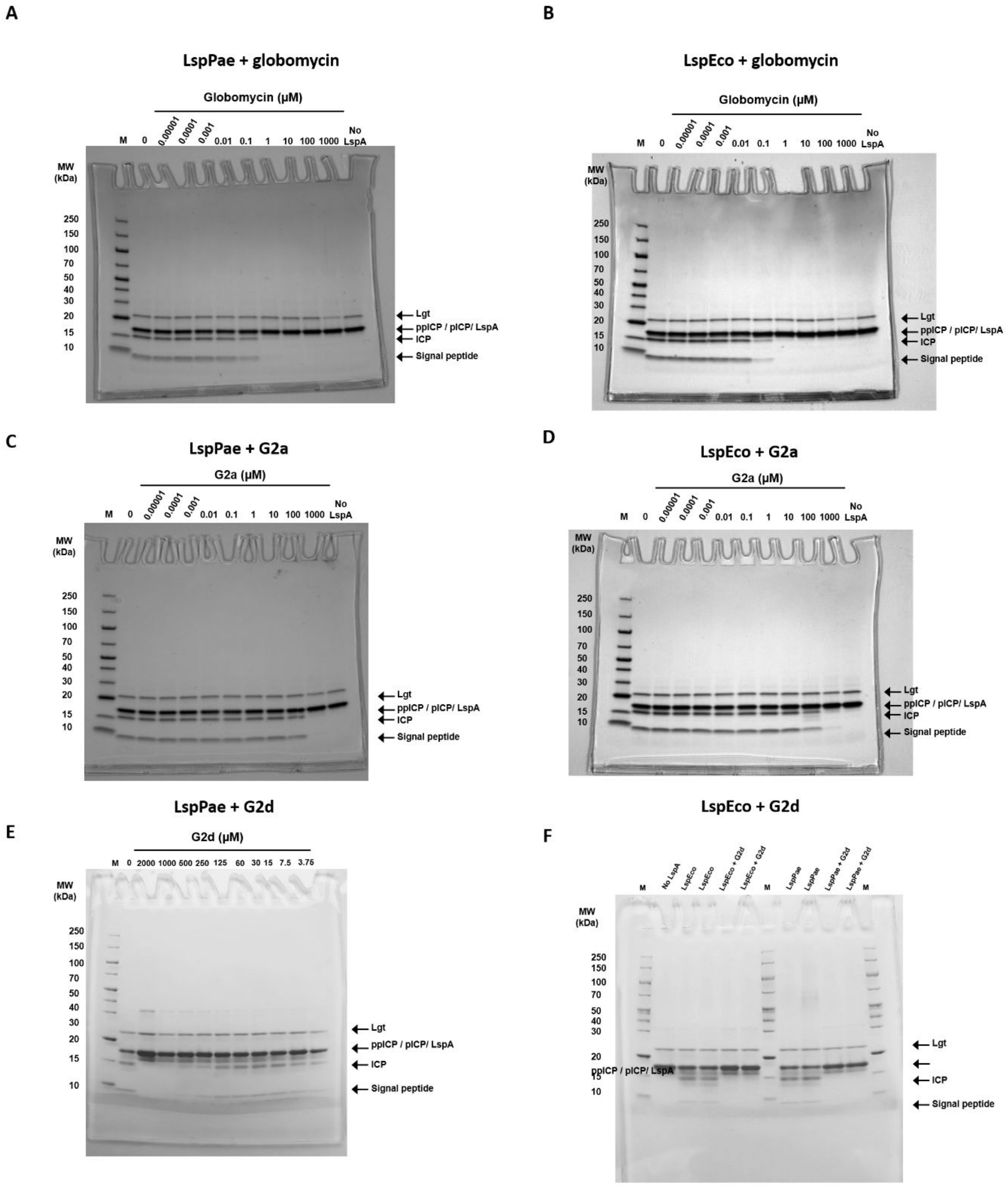

Figure S14. *Pa*LspA and *Ec*LspA gel-shift inhibition assays by globomycin, G2a and G2d. (A) *Pa*LspA inhibition by globomycin. **(B)** *Ec*LspA inhibition by globomycin. **(C)** *Pa*LspA inhibition by G2a. **(D)** *Ec*LspA inhibition by G2a. **(E)** *Pa*LspA inhibition by G2d. **(F)** *Ec*LspA and *Pa*LspA inhibition by G2d. Full assay details are described under Methods. Pre-proICP concentration was 12 µM, DOPG concentration was 600 µM, Lgt concentration was 1.2 µM. The Lgt catalysed reaction was allowed to proceed for 60 min at 37 ^{o}C. Inhibitor (globomycin, G2a or G2d) was added and the LspA reaction initiated by addition of 100 nM LspA. The LspA reaction was allowed to proceed for 30 min at 37 ^{o}C.

References

1. Vogeley, L.; El Arnaout, T.; Bailey, J.; Stansfeld, P. J.; Boland, C.; Caffrey, M. Structural basis of lipoprotein signal peptidase II action and inhibition by the antibiotic globomycin. *Science*. **2016**, 351, 876-880.

2. Olatunji Olatunji, S.; Yu, X.; Bailey, J.; Huang, C. Y.; Zapotoczna, M.; Bowen, K.; Remškar, M.; Müller, R.; Scanlan, E. M.; Geoghegan, J. A.; Olieric, V.; Caffrey, M. Structures of Lipoprotein Signal Peptidase II from Staphylococcus Aureus Complexed with Antibiotics Globomycin and Myxovirescin. *Nat. Commun.* **2020**, 11, 140.

3. Wiegand, L; Hilpert, K; Hancock, R.E.W. Agar and broth dilution methods to determine the minimal inhibitory concentration (MIC) of antimicrobial substances, *Nat. Protoc* **2008**, 3, 163-175.

4. Hosseinzadeh, P.; Bhardwaj, G.; Mulligan, V.K.; Shortridge, M. D.; Craven, T. W.; Pardo-Avila, F.; Rettie, S. A.; Kim, D. E.; Silva, D. A.; Ibrahim, Y. M.; Webb, I. K.; Cort, J. R.; Adkins, J. N.; Varani, G.; Baker, D. Comprehensive computational design of ordered peptide macrocycles. *Science*. **2017**, 358, 1461-1466.

5. Mulligan, V. K.; Workman, S.; Sun, T.; Rettie, S.; Li, X.; Worrall, L. J.; Craven, T. W.; King, D. T.; Hosseinzadeh, P.; Watkins, A. M.; Renfrew, P. D.; Guffy, S.; Labonte, J. W.; Moretti, R.; Bonneau, R.; Strynadka, N. C. J.; Baker, D. Computationally designed peptide macrocycle inhibitors of New Delhi metallo-β-lactamase 1. *Proc. Natl. Acad. Sci.* **2021**, 118, e2012800118.

MS Spectra for Generation 1 Peptides:

G1a: [M+H]+ calculated 655.44 Da, found 655.54 Da.

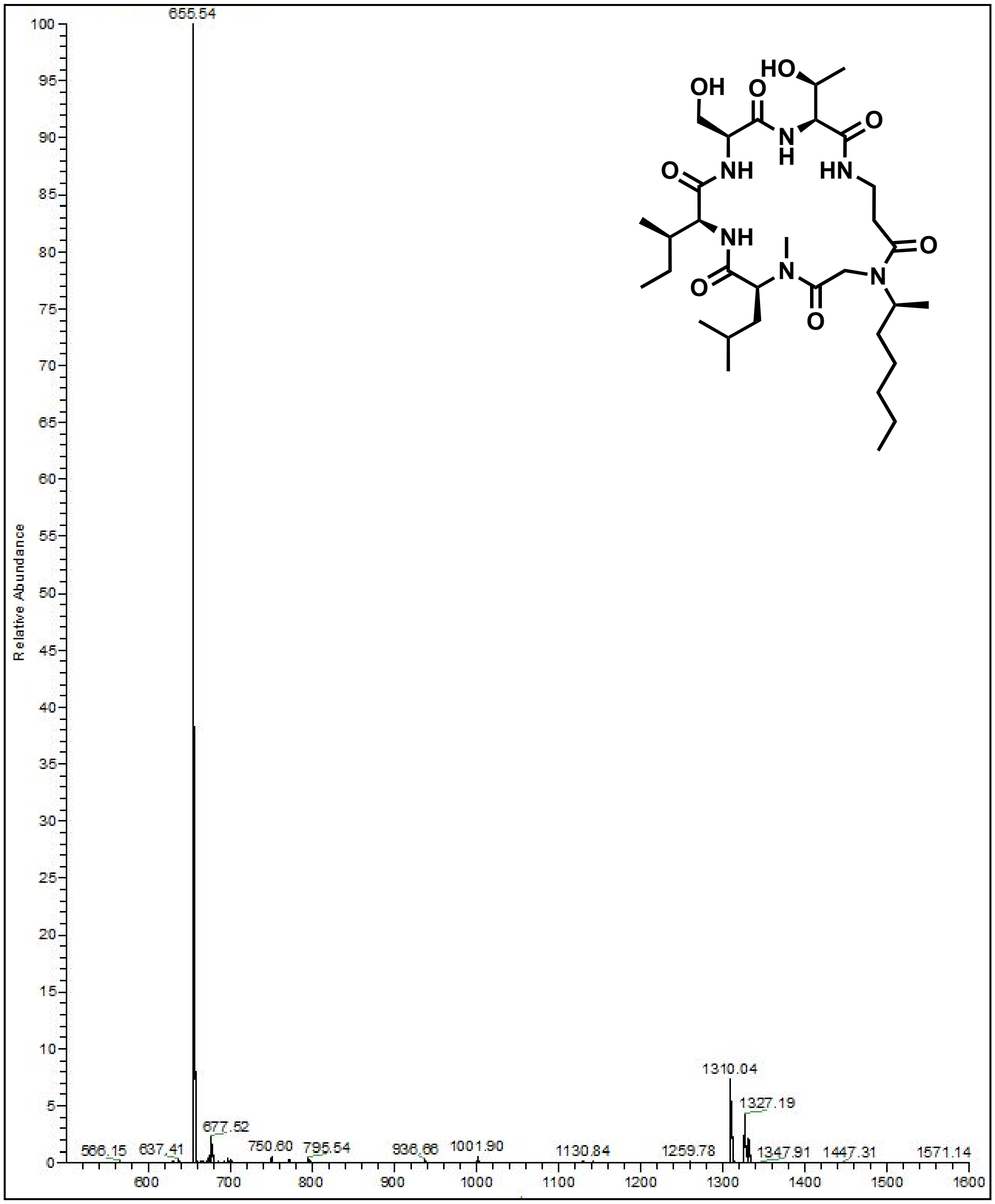

G1b: [M+H]+ calculated 655.44 Da, found 655.44 Da.

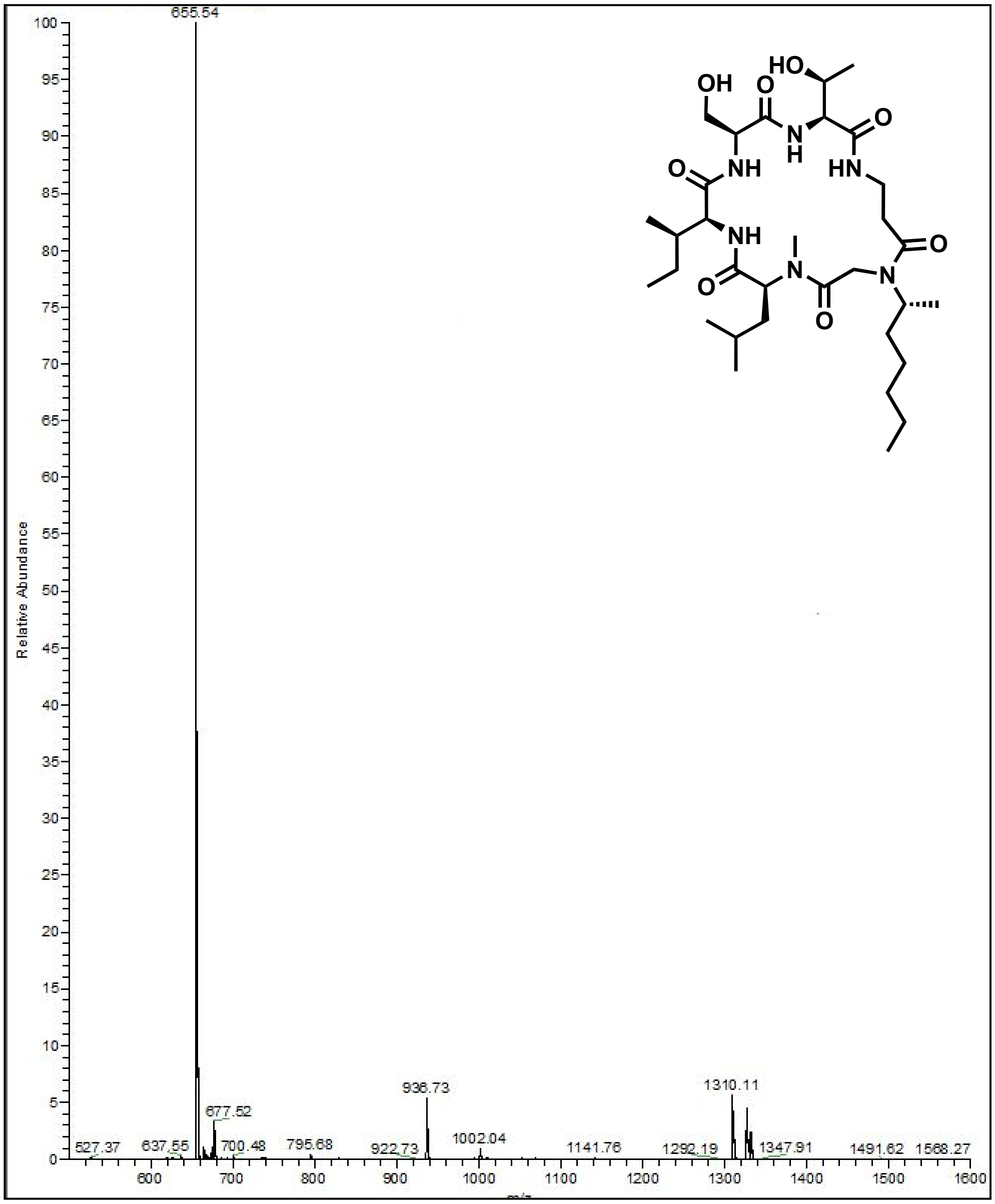

G1c: [M+H]+ calculated 669.54 Da, found 669.54 Da.

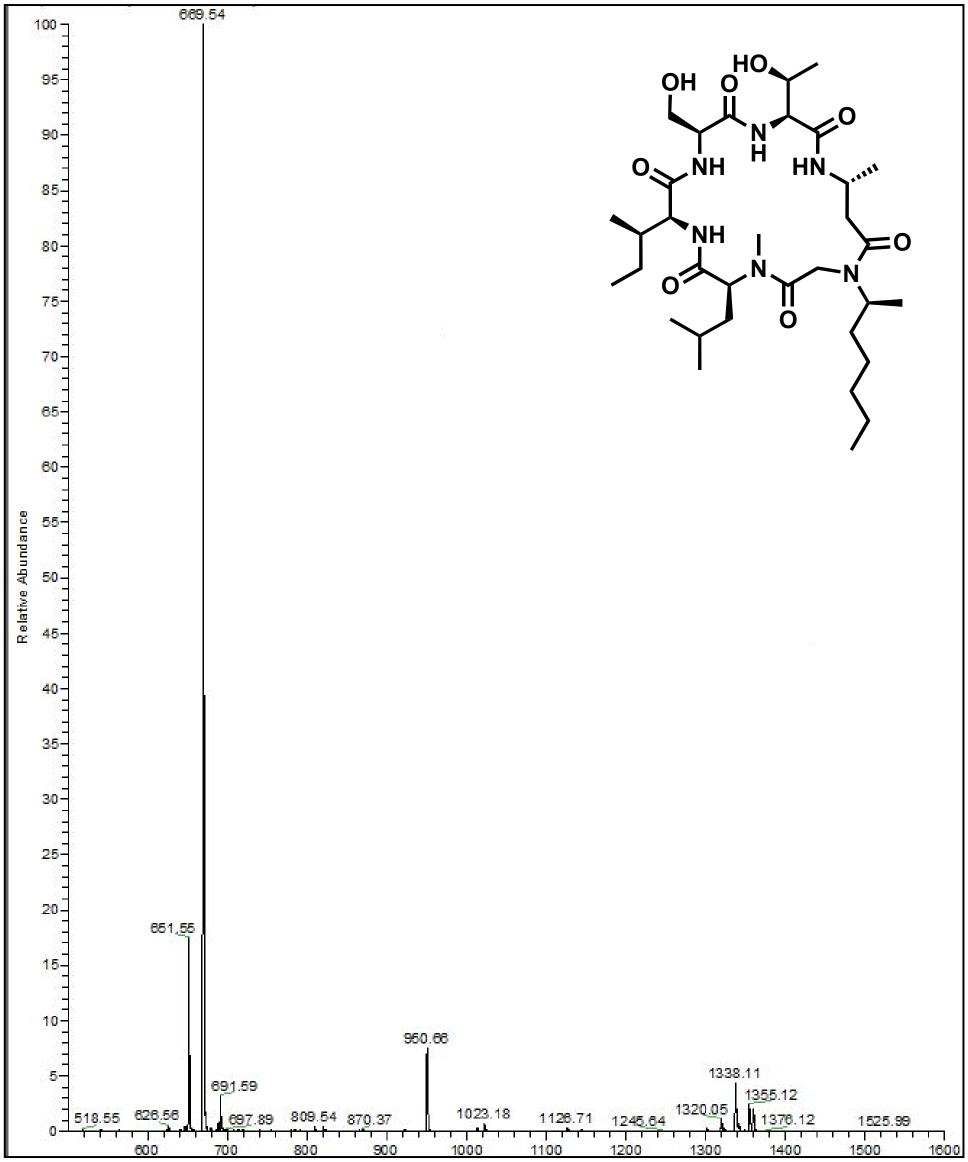

G1d: [M+H]+ calculated 669.45 Da, found 669.54 Da.

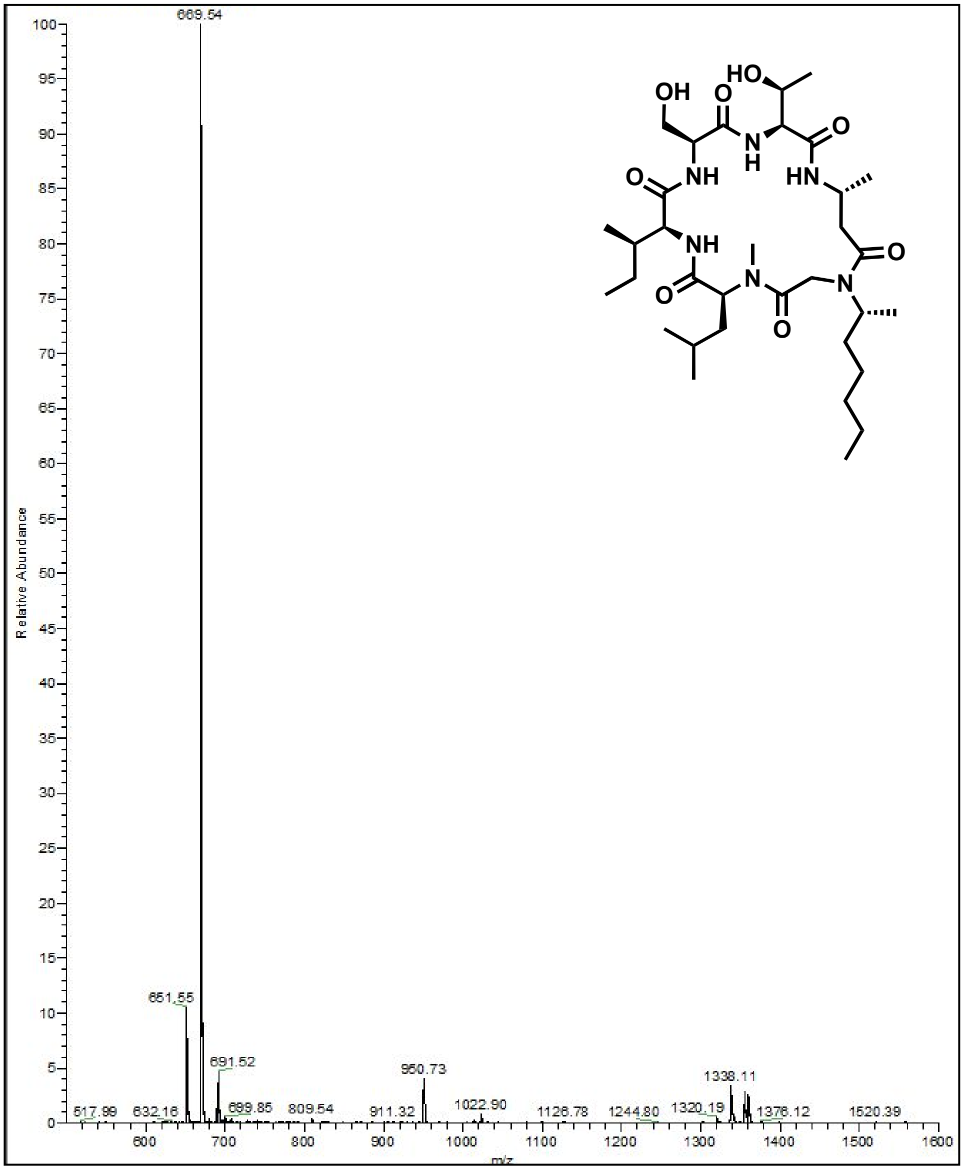

G1e: [M+H]+ calculated 669.45 Da, found 669.54 Da.

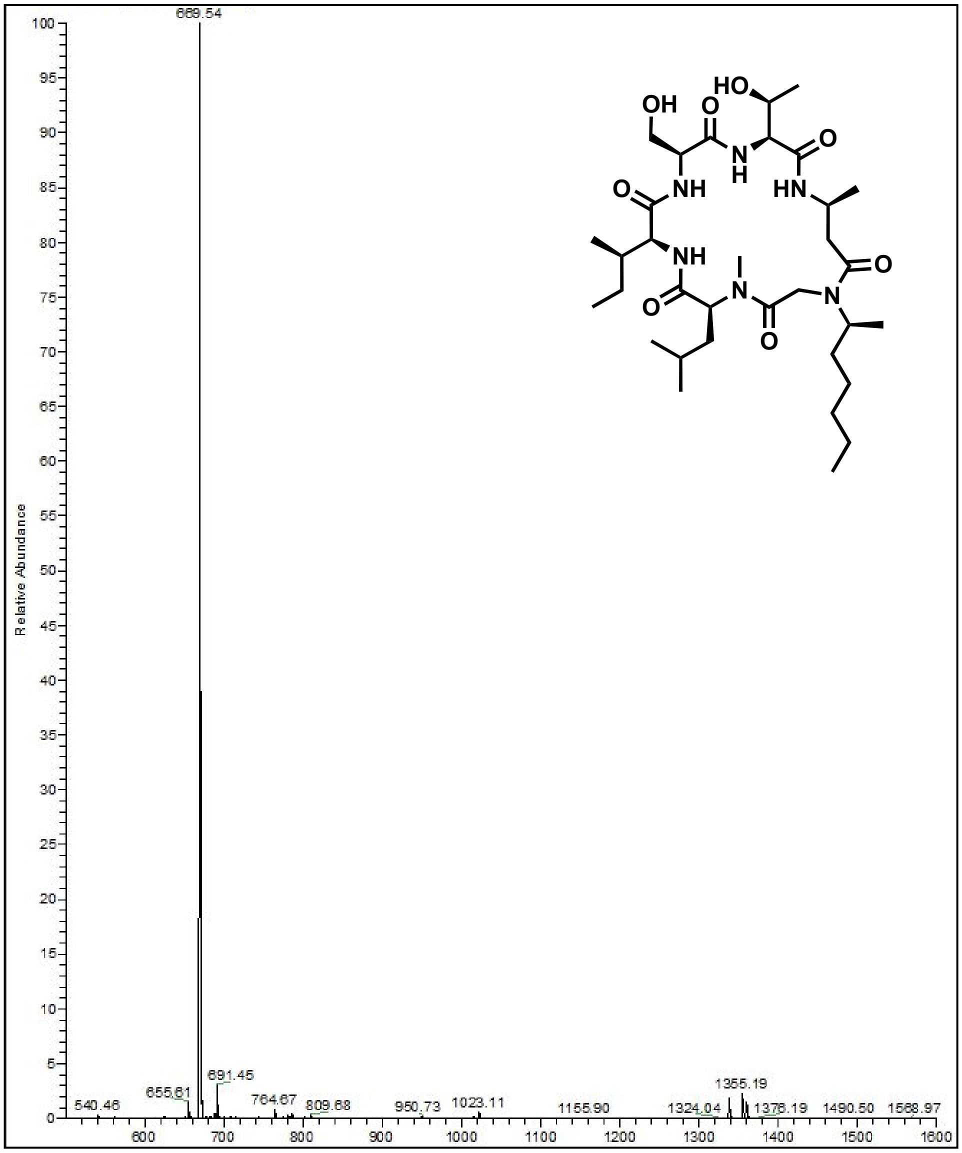

G1f: [M+H]+ calculated 669.45 Da, found 669.54 Da.

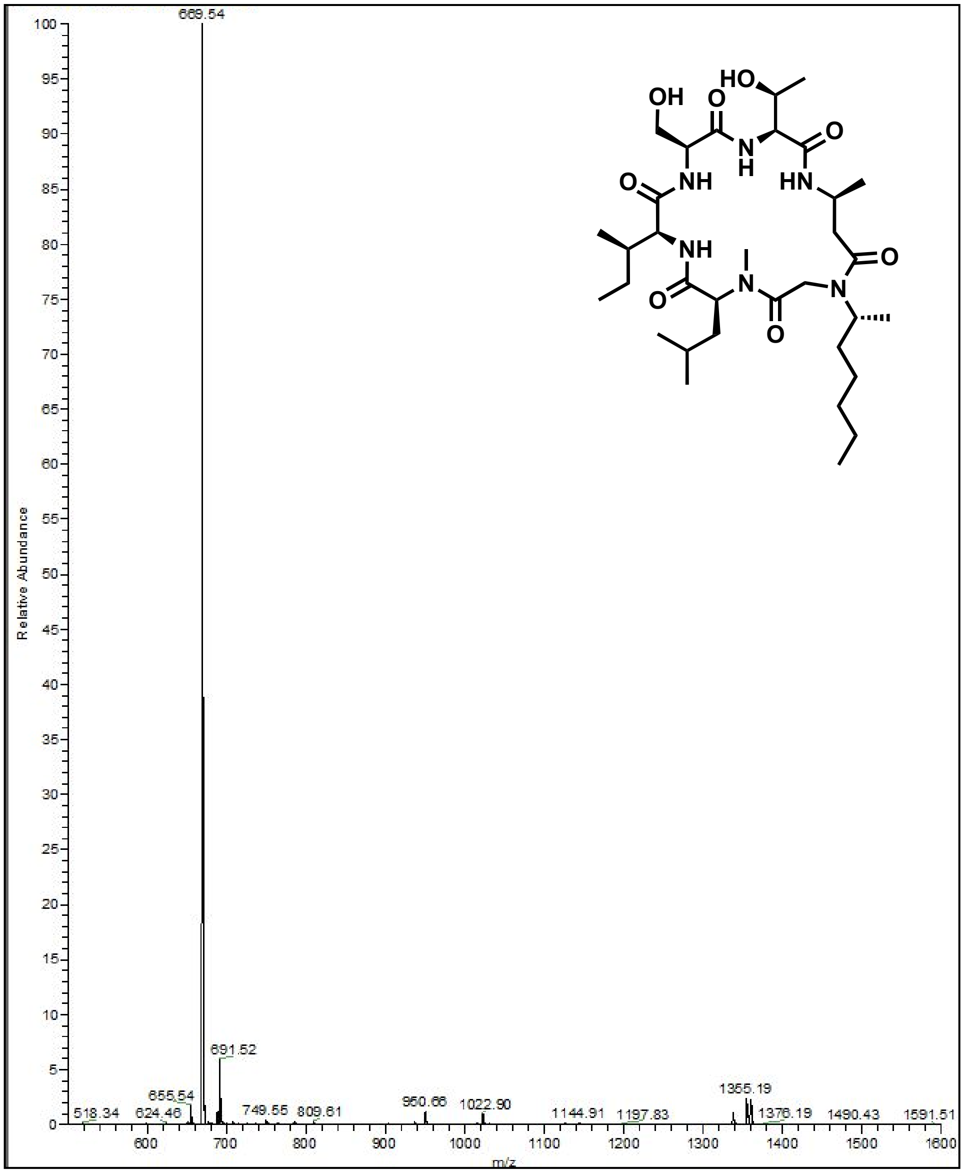

MS Spectra for Generation 2 Peptides:

G2a: [M+H]+ calculated 683.47 Da, found 683.47 Da.

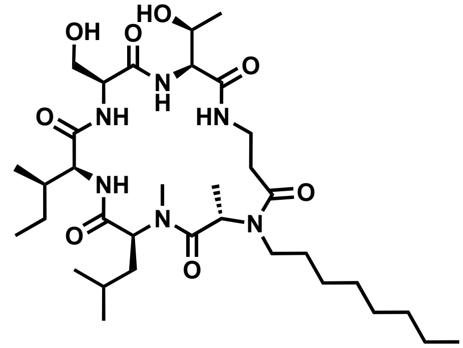

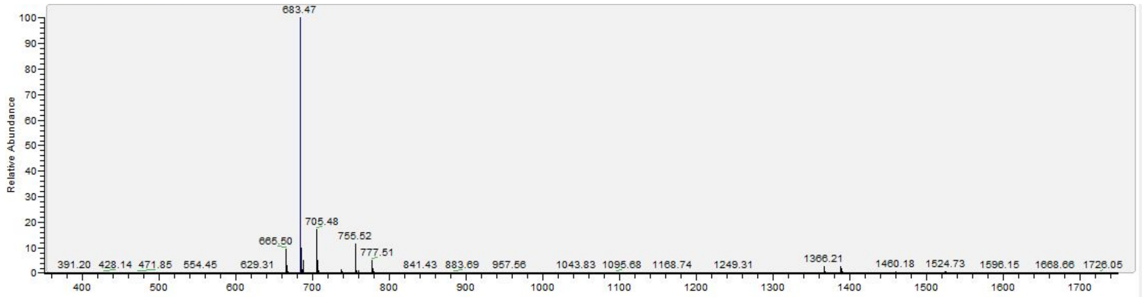

G2b: [M+H]+ calculated 683.47 Da, found 683.48 Da.

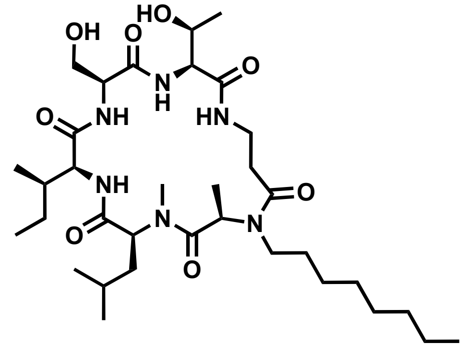

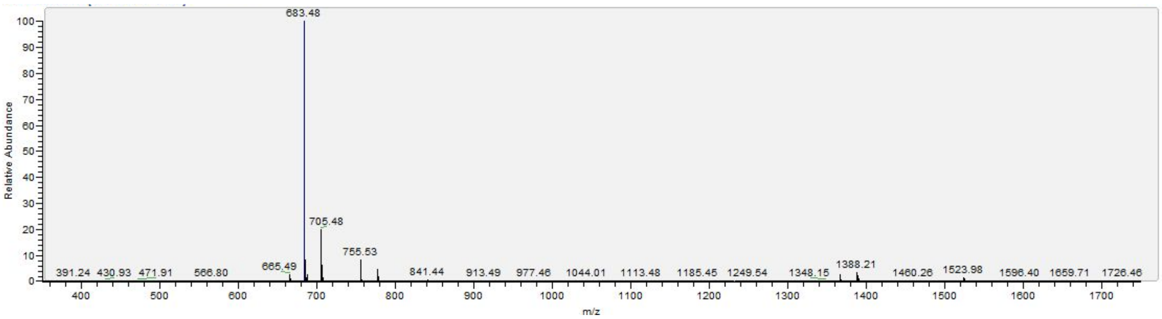

G2c: [M+H]+ calculated 669.45 Da, found 669.46 Da.

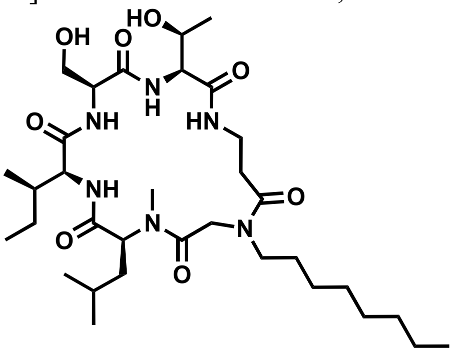

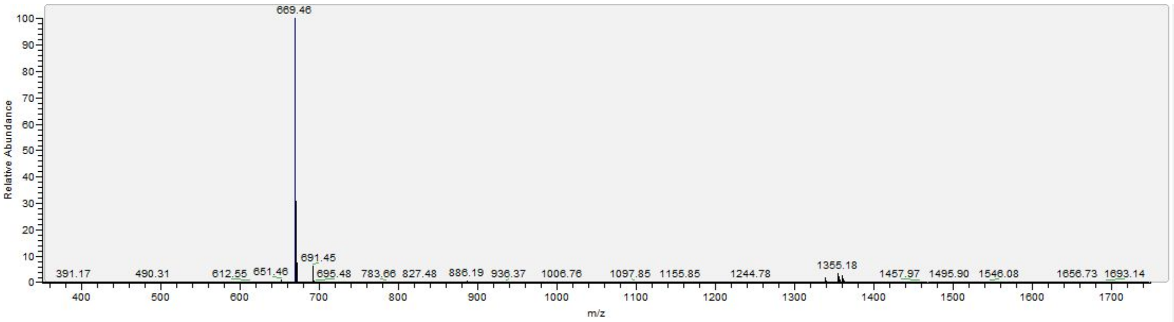

G2d: [M+H]+ calculated 725.52 Da, found 725.54 Da.

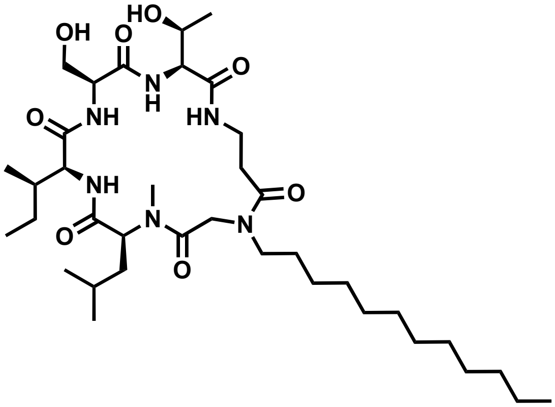

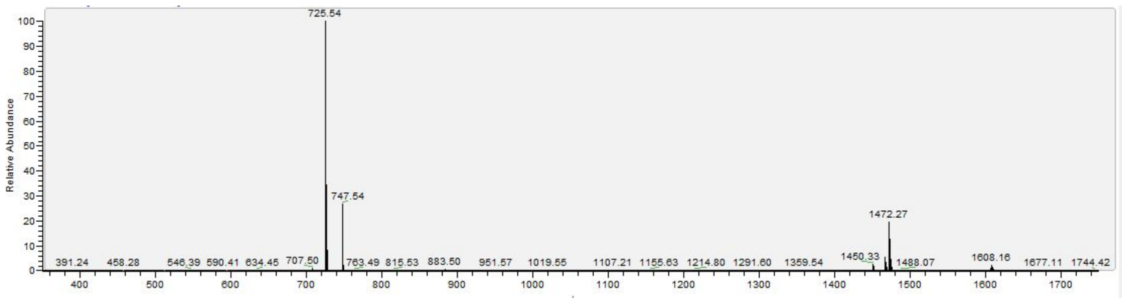

G2e: [M+H]+ calculated 717.44 Da, found 717.46 Da.

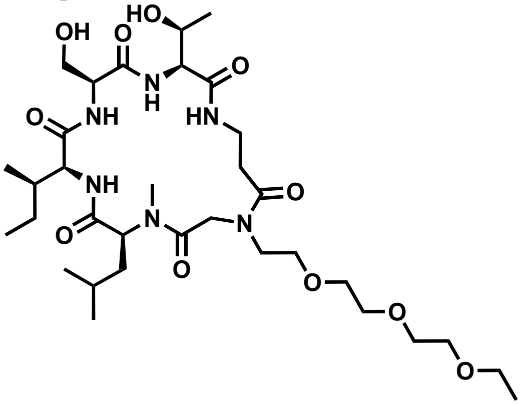

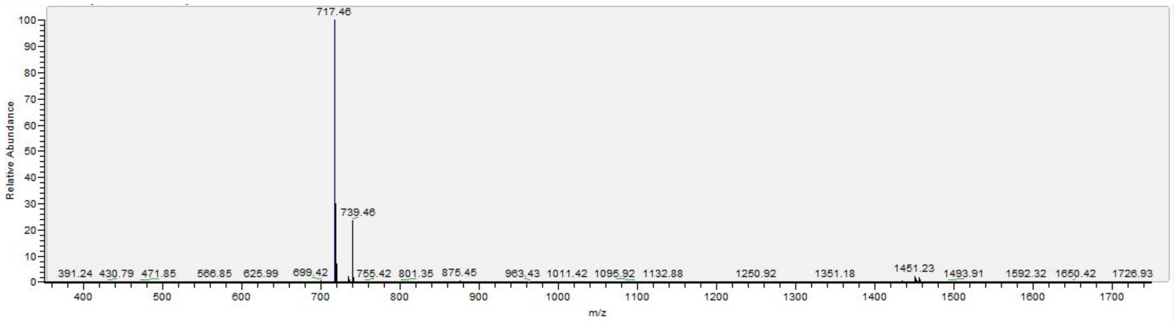

G2f: [M+H]+ calculated 923.55 Da, found 923.62 Da.

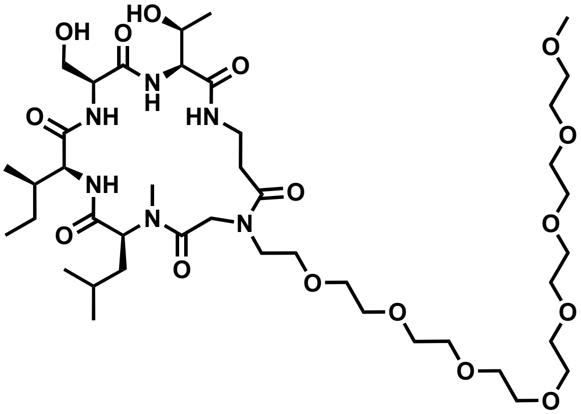

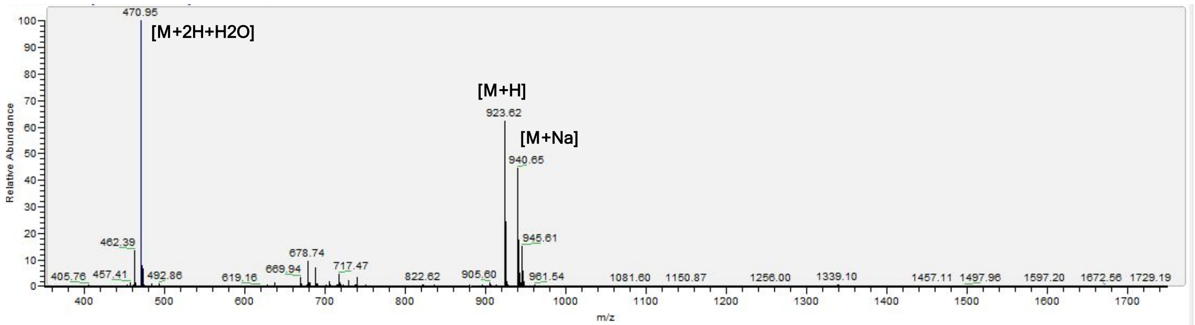

Supplement: Supplementary file 1 — cb4c00076_si_001.pdf [file cb4c00076_si_001.pdf]
